# Expanded inverted repeat region with large scale inversion in the first complete plastid genome sequence of *Plantago ovata*

**DOI:** 10.1038/s41598-020-60803-y

**Published:** 2020-03-03

**Authors:** Sajjad Asaf, Abdul Latif Khan, Lubna, Adil khan, Arif Khan, Gulzar Khan, In-Jung Lee, Ahmed Al-Harrasi

**Affiliations:** 1 Natural and Medical Sciences Research Center, University of Nizwa, Nizwa, Oman; 2 Department of Botany, Garden Campus, Abdul Wali Khan University Mardan, Khyber Pakhtunkhwa, Pakistan; 3 Genomics Group, Faculty of Biosciences and Aquaculture, Nord University, Bodø, 8049 Norway; 4 Institute for Biology and Environmental Sciences, Carl von Ossietzky University, Oldenburg, Oldenburg Germany; 5 School of Applied Biosciences, Kyungpook National University, Daegu, 41566 Republic of Korea

**Keywords:** Plant genetics, Comparative genomics

## Abstract

*Plantago ovata* (Plantaginaceae) is an economically and medicinally important species, however, least is known about its genomics and evolution. Here, we report the first complete plastome genome of *P. ovata* and comparison with previously published genomes of related species from Plantaginaceae. The results revealed that *P. ovata* plastome size was 162,116 bp and that it had typical quadripartite structure containing a large single copy region of 82,084 bp and small single copy region of 5,272 bp. The genome has a markedly higher inverted repeat (IR) size of 37.4 kb, suggesting large-scale inversion of 13.8 kb within the expanded IR regions. In addition, the *P. ovata* plastome contains 149 different genes, including 43 tRNA, 8 rRNA, and 98 protein-coding genes. The analysis revealed 139 microsatellites, of which 71 were in the non-coding regions. Approximately 32 forward, 34 tandem, and 17 palindromic repeats were detected. The complete genome sequences, 72 shared genes, *matK* gene, and *rbcL* gene from related species generated the same phylogenetic signals, and phylogenetic analysis revealed that *P. ovata* formed a single clade with *P. maritima* and *P. media*. The divergence time estimation as employed in BEAST revealed that *P. ovata* diverged from *P. maritima* and *P. media* about 11.0 million years ago (Mya; 95% highest posterior density, 10.06–12.25 Mya). In conclusion, *P*. *ovata* had significant variation in the IR region, suggesting a more stable *P*. *ovata* plastome genome than that of other Plantaginaceae species.

## Introduction

Chloroplasts carry out photosynthesis along with other crucial functions including the biosynthesis of starch, fatty acids, amino acids, and pigments in plants and various other eukaryotic lineages^1,2^. Typically, maternally inherited angiosperm plastome genomes are extremely conserved and have a quadripartite structure ranging from 115 to 165 kb in length and comprising a small-single-copy region (SSC) and large-single-copy region (LSC) parted by an inverted repeat (IR)^3,4^. The gene order and content are mostly conserved; however, at genome and gene levels, a number of variations have been reported in various plastome genomes^5^. The plastome genome is a useful tool in evolutionary studies and genomics because of its non-recombinant nature^6^. Furthermore, the plastome genome is highly conserved mostly in angiosperms, evolutionary hotspots such as addition and deletion of genes^7^, contraction and expansion of IR region^8^, inversion of various genomic regions, copy number variations in tandem repeats^9^, and single nucleotide polymorphisms (SNPs)^7^. SNPs and InDels are valuable molecular markers in these conserved plastomes within the species level^10^. In brief, various characteristics including uniparental inheritance, absence of recombination, and a haploid state^11,12^ make plastome genome valuable for phylogeographic and phylogenetic studies to understand the history of most plant lineages^13,14^. At present, there are over 2700 plastid genome sequences submitted to the National Centre for Biotechnology Information (NCBI), including all of the major plant lineages. Altogether, plastome genome sequences possess sufficient information, and sequencing of these genomes play a vital role in diversification and to facilitate comparisons among various plant species^15,16^.

*Plantago* is an important genus of Plantaginaceae^17^, commonly known as Plantains and is usually a perennial or annual herb or sub-shrub. The genus has a worldwide distribution with ca. 200 species, among which only two species namely *P. psyllium* and *P. ovata* have been widely studied for seed husk production^18^. Morphologically, the seed husk of *P. ovata* is enclosed by a thin white membrane, usually known as *Blonde Psyllium* in English. The genetic diversity of *Plantago* species is important for breeding various cultivars, which are valuable additions that are resistant to different diseases^19^. The meiotic system and chromosomal structure of *P. ovata* have various features which account for its narrow genetic base such as a small 621-Mb genome size with 4 (2n = 2x = 8) chromosomes^20^.

In an comprehensive phylogenetic study of the family Plantaginaceae based on different chemical, embryological, and morphological data, researchers were unable to identify a sister-family for this monophyletic group^17^. However, Dhar *et al*.^21^ and Ronsted *et al*.^22^ have determined various molecular markers that are useful for inferring the phylogenetic relationships of the genus *Plantago*. The ITS and 5S rRNA regions depicted on the sequence data showed that *P. ovata* is closely related to *P. arenaria*^23^. In recent time with the advancement in molecular techniques, different molecular markers were used to access the intra and interspecific relationship among *Plantago* species^19,24^. However, such studies investigated smaller intraspecific diversity than the interspecific diversity.

Taking into account the taxonomic and phylogenetic complications for the genus, and lack of concentrated evidences, here in the present study, we sequenced and performed a comparative analysis of the complete plastome of *P*. *ovata* with the plastomes of six related species (*P. media, P. maritima, Veronica nakaiana*, *V. presica, V. veronicstrum*, and *Digitalis lanata*). We aimed to elucidate and compare the regions of high sequence divergence, IR expansion, intron contents, and phylogenomics of *P. ovata* with plastomes of related species. In addition, we employed divergence time estimation to reconstruct the phylogenetic relationship and divergence time of *P. ovata*, *P. media*, and *P. maritima* based on whole plastomes.

## Results

### General features of *P. ovata* plastome sequence and its comparison with related species

**Figure 1:**
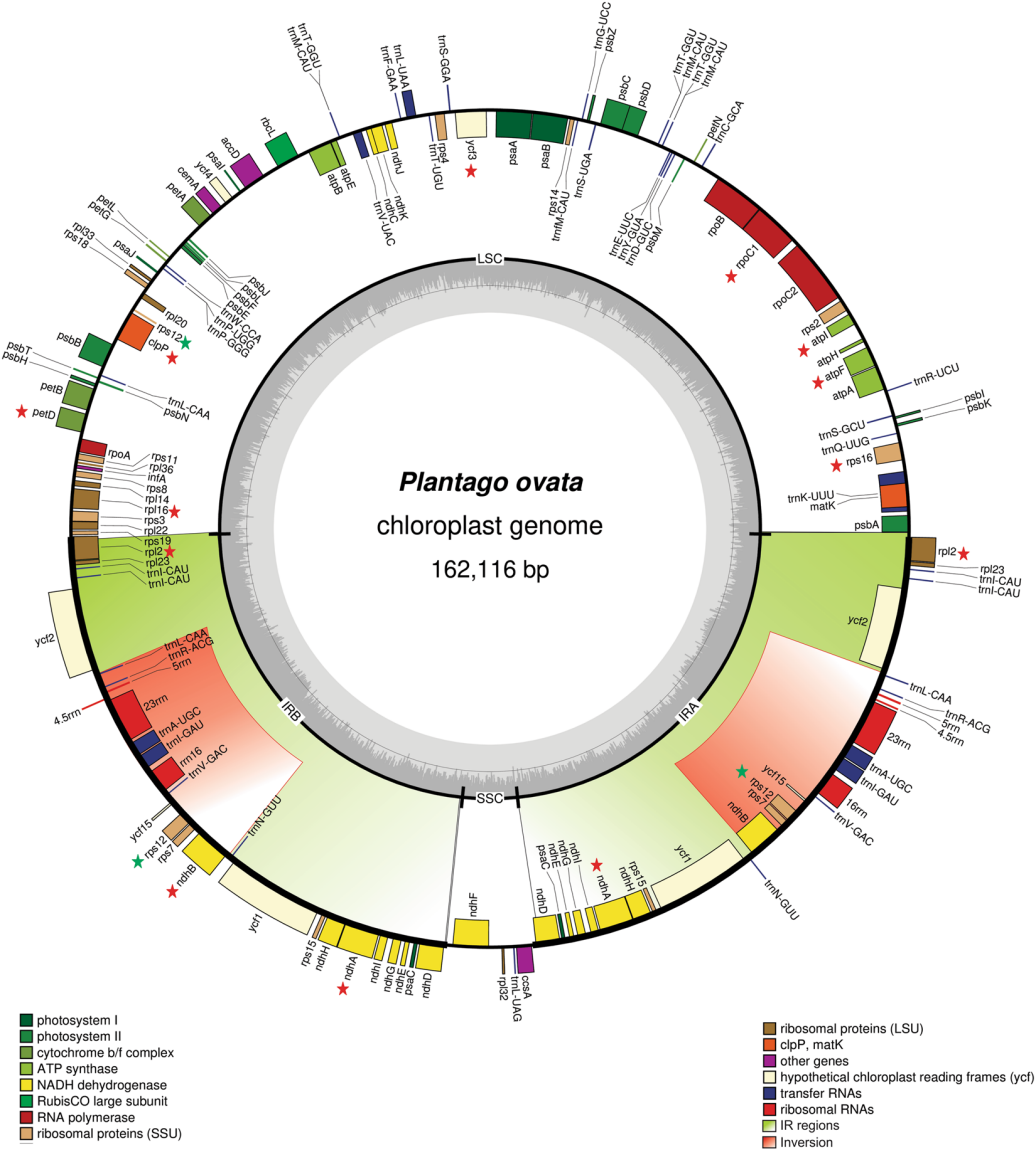
Gene map of the *P. ovata* plastome genome. Genes drawn inside the circle are transcribed clockwise, and those outside the circle are transcribed counterclockwise. The red and green colour asterisks indicate intron-containing and trans-spliced genes respectively. Genes belonging to different functional groups are colour-coded. The darker grey in the inner circle corresponds to GC content, and the lighter grey corresponds to AT content.

The genome size of *P. ovata* plastome is 162,116 bp and has a typical quadripartite structure containing an LSC (82,084 bp) and SSC (5,272 bp) and separated by a pair of identical IRs (37,380 bp) each. The GC content (38.1%) and LSC region of *P. ovata* is consistent with the previously reported plastomes belonging to the family Plantaginaceae (Table 1). Compared to the typical angiosperm genome structure, for example, that of *P. media* and *P. maritima*, *P. ovata* had considerably higher number of IRs measuring up to 37.4 kb in size (Table 1, Fig. 1) than other angiosperms. Additionally, in all the sequenced plastomes from Plantaginaceae, *P. ovata* has the largest plastome, except *P. media* (164,130 bp; Table 1). The plastome of *P. ovata* contains 147 different genes, including 43 tRNA, 8 rRNA, and 96 protein-coding genes (12 small and 9 large ribosomal subunits, 4 DNA-dependent RNA polymerases, 33 photosynthesis-related proteins, and 10 genes encoding other proteins) (Table 2, Fig. 1). About, 15.40% of the functional genes contains introns, including 7 tRNA and 16 protein-coding genes; the two genes *clpP* and *ycf3* contain two introns (Table 3, Fig. 1). The lengths of these introns range from 483 bp (*trnV-UAC*) to 2,434 bp (*trnK-UUU*). The *rps12* gene (small ribosomal protein 12) is trans-spliced and contains one intron; furthermore, its 5′ end exon is located in the LSC region, whereas the 3′ end exon is located in the IRb regions and also duplicated in the IRa region (Fig. 1).

**Table 1:** Summary of complete chloroplast genomes.

	Plantago ovata	Plantago media	Plantago maritima	Veronica nakaiana	Veronica persica	Veronicstrum sibiricum	Digitalis lanata
Size (bp)	162,116	164,130	158,358	152319	150198	152930	153108
Overall GC contents	38.1	38.0	38.6	37.9	37.9	38.3	38.6
LSC size in bp	82084	82757	82222	83194	81849	83615	83934
SSC size in bp	5272	4577	8665	17702	17419	17801	17688
IR size in bp	37380	38398	33736	25711	25465	25757	25743
Protein coding regions size in bp	76904	88383	85374	80376	79587	80142	78693
tRNA size in bp	3211	2871	2942	2798	3153	2803	2777
rRNA size in bp	9048	9062	9058	9051	9051	9050	9052
Number of genes	149	140	137	133	130	131	130
Numebr of protein coding genes	98	94	90	88	86	86	85
Numebr of rRNA	8	8	8	8	8	8	8
Number of tRNA	43	38	39	37	36	37	37
Genes with introns	16	16	13	15	14	15	15

**Table 2:** Genes in the sequenced *P. ovata* chloroplast genome.

Category	Group of genes	Name of genes
Self-replication	Large subunit of ribosomal proteins	rpl2^*,a^, 14, 16^*^, 20, 22, 23^a^, 32, 33, 36
	Small subunit of ribosomal proteins	*rps2, 3, 4, 7*^a^, 8, 11, 12^*,a^*, 14, 15*^a^,16^*^, 18, 19
	DNA dependent RNA polymerase	*rpoA, B* ^*a*^, C1^*^, C2
	rRNA genes	*rrn 4.5, rrn 5, rrn 16, rrn23*
	tRNA genes	trnA-UGC^*^, trnC-GCA, trnD-GUC, trnE-UUC trnF-GAA, trnfM-CAU, trnG-UCC, trnH-GUG, trnI-CAU, trnI-GAU^*^, trnK-UUU^*^, trnL-CAA, trnL-UAA^*^, trnL-UAG, trnM-CAU, trnN-GUU, trnP-GGG, trnP-UGG, trnQ-UUG, trnR-ACG, trnR-UCU, trnS-GCU, trnS-GGA, trnS-UGA, trnT-GGU, trnT-UGU, trnV-GAC, trnV-UAC^*^, trnW-CCA, trnY-GUA
Photosynthesis	Photosystem I	*psaA, B, C*^a^*, I, J*,
	Photosystem II	*psbA, B, C, D, E, F, H, I, J, K, L, M, N, T, Z*
	NadH oxidoreductase	NdhA^*,a^, B^*,a^*, C, D*^a^*, E*^a^*, F, G*^a^*, H*^a^*, I*^a^*, J, K*
	Cytochrome b6/f complex	*petA, B*^a^, D^*^, G, L, N
	ATP synthase	atpA, B, E, F^*^, H, I
	Rubisco	*rbcL*
Other genes	Maturase	*matK*
	Protease	clpP^*^
	Envelop membrane protein	*cemA*
	Subunit Acetyl- CoA-Carboxylate	*accD*
	c-type cytochrome synthesis gene	*ccsA*
Unknown	Conserved Open reading frames	*ycf1*^a^, 2^*,a^, 3^*^, 4, 15^a^

^*^Genes containing introns; ^a^Duplicated gene (Genes present in the IR regions).

**Table 3:** The genes with introns in the *P. ovata* chloroplast genome and the length of exons and introns.

Gene	Location	Exon I (bp)	Intron 1 (bp)	Exon II (bp)	Intron II (bp)	Exon III (bp)
*atpF*	LSC	141	706	411		
*petB*	LSC	6	715	642		
*petD*	LSC	12	693	474		
*rpl2*	IR	391	676	438		
*rpl16*	LSC	9	1602	393		
*rps16*	LSC	40	862	227		
*rpoC1*	LSC	453	741	1611		
*rps12*		114	—	232	535	26
*clpP*	LSC	69	727	291	567	237
*ndhA*	IR	552	1073	531		
*ndhB*	IR	726	675	753		
*ycf3*	LSC	124	713	230	740	150
*trnA-UGC*	IR	38	815	35		
*trnI –GAU*	IR	42	805	35		
*trnL-UAA*	LSC	37	507	50		
*trnK -UUU*	LSC	37	2434	35		
*trnV-UAC*	LSC	37	483	37		

Overall, the protein coding, rRNA and tRNA genes contain 47.96%, 5.57%, and 1.97%, respectively, of the *P. ovata* plastome sequence (Table S1). The GC content for tRNA (52.10%) and rRNA (55.20%) are the highest, followed by protein-coding genes (39%) in the coding regions. Similarly, GC content within the protein-coding genes at the first, second, and third positions of codon is 55.60%, 47.40%, and 36.70%, respectively (Table S1). Codon usage and codon–anticodon recognition pattern of *P. ovata* plastome are summarised in Table S2, in which a total of 24,322 codons from a genome size of 72,968 bp have been represented. Based on tRNA and protein-coding genes, RSCU frequency was calculated (Table S1). The most common amino acid was leucine (10.90%), whereas the least common one was cysteine (1.30%, Table S2). Codon usage is biased towards a high representation of A and T at the third position (82.60%), revealing a pattern similar to angiosperm plastid genomes (Table S1).

### Comparative analysis of *P. ovata* plastome with the plastomes of related species

**Figure 2:**
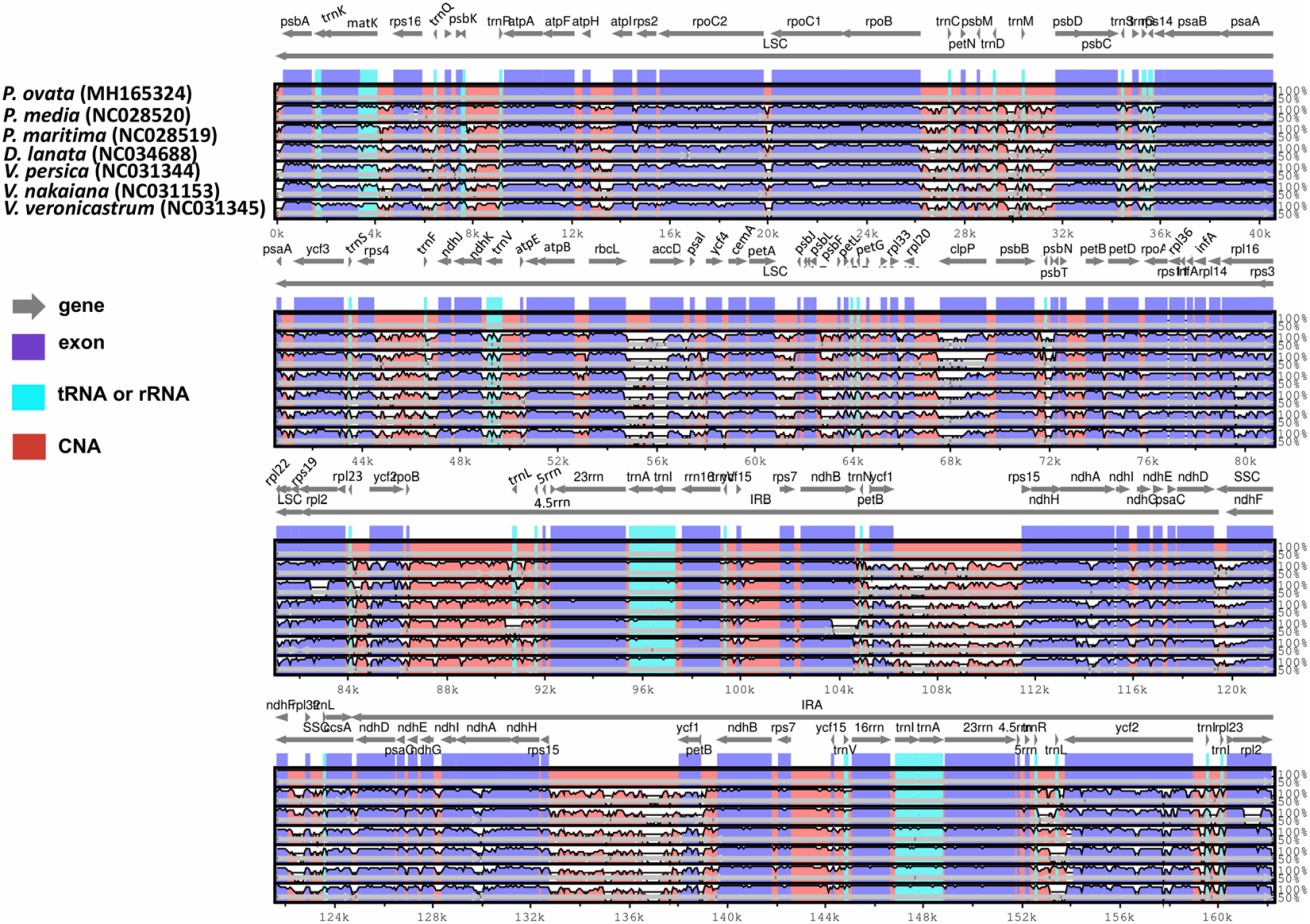
Alignment visualization of the *P. ovata* plastome genome sequences. VISTA-based identity plot showing sequence identity among the seven-species using *P. ovata* as a reference. The vertical scale indicates percent identity, ranging from 50% to 100%. The horizontal axis indicates the coordinates within the chloroplast genome. Arrows indicate the annotated genes and their transcription direction. The thick black lines show the inverted repeats (IRs).

**Figure 3:**
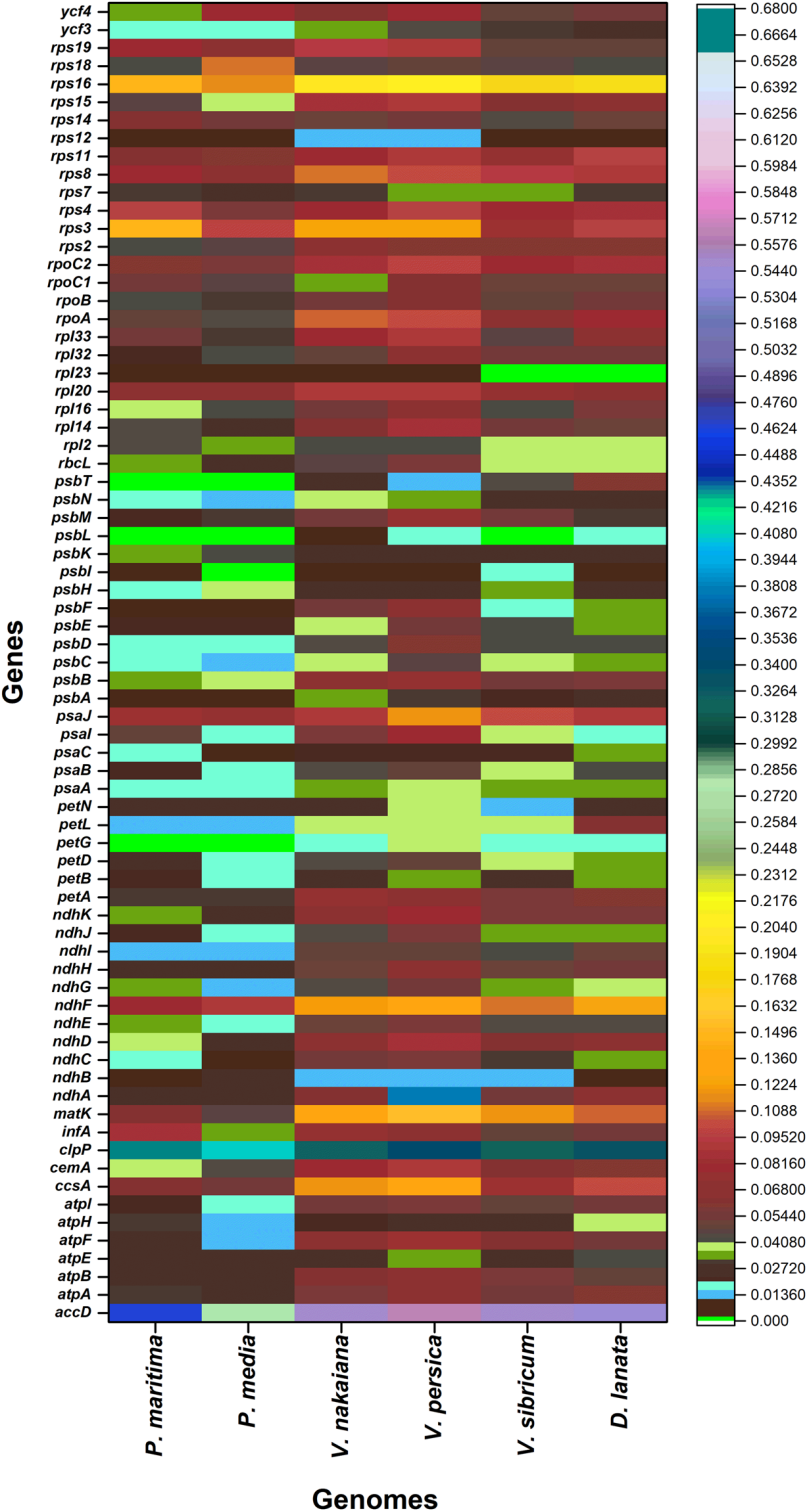
Pairwise sequence distance of *P. ovata, 72* genes with related species.

The synteny of *P. ovata* plastomes with seven other species from Plantaginaceae was analysed by mVISTA. The results showed high sequence similarities among the plastomes of several species, especially in protein-coding and IR regions (Fig. 2). The highest level of divergence was detected in intergenic regions, including *atpH-atpI*, *rpoC1-rpoC2*, *ycf1-rps15*, *accD-psaI*, *psaA-ycf3*, and *trnL*-*rrn5*. Besides these regions, some divergence was observed in protein-coding genes, viz. *accD, clpP, ndhA, ndhF, rpl16, petD, matK, rpl16, ycf2, ycf1*, and *rpl2* (Fig. 2). In a pairwise sequence divergence analysis, *P. ovata* exhibited highest divergence (0.20) with *V. persica* and showed lowest divergence with *P. media* (0.048) (Table S3). The most divergent genes were *clpP*, *accD*, *psaJ, rps3*, *ccsA*, and *matK*. The highest pairwise divergence was detected in *clpP* gene (0.67) and *accD* gene (0.56) (Fig. 3). Furthermore, the synonymous (Ks) and non-synonymous (Ka) values of plastomes were calculated. The results revealed that *P. ovata* exhibited highest Ka/Ks value of plastome with *V*. *nakiana* (0.198/0.2506) and lowest with *P*. *maritima* (0.05/0.06), respectively. However, the most divergent genes, *accD* and *clpP*, showed variable results. The highest Ka/Ks value was exhibited by *V. nakiana* for *accD* gene, whereas the highest Ka/Ks value was exhibited by *P. maritima* for *clpP* gene (Figure S1). The length of the most divergent gene, *accD*, was 1,356 bp (452 aa), 1,347 bp (449 aa), and 1,257 bp (425 aa) in *P. ovata*, P. *media*, and *P. maritima*, respectively. In the other four species, the length of *accD* gene ranged from 1,470 to 1,497 bp (Fig. S2). Variation in the intron content of *clpP* gene was found in *P. maritima*, in which there was a complete loss of both introns (Fig. S3). This forms the basis for the highest divergence of *P. maritima* with *P. ovata* genome for the *clpP* gene. A comparative analysis of the *P. ovata* plastome revealed a varied number of SNPs and InDel substitution. The highest number of SNPs was detected in *V. persica* (53,660), whereas the lowest number of SNPs was observed in *P. maritima* (16,386). The highest number of InDel substitutions was detected in *P. maritima* (74,448) plastome (Table S4).

### Simple sequence repeat (SSR) analysis

**Figure 4:**
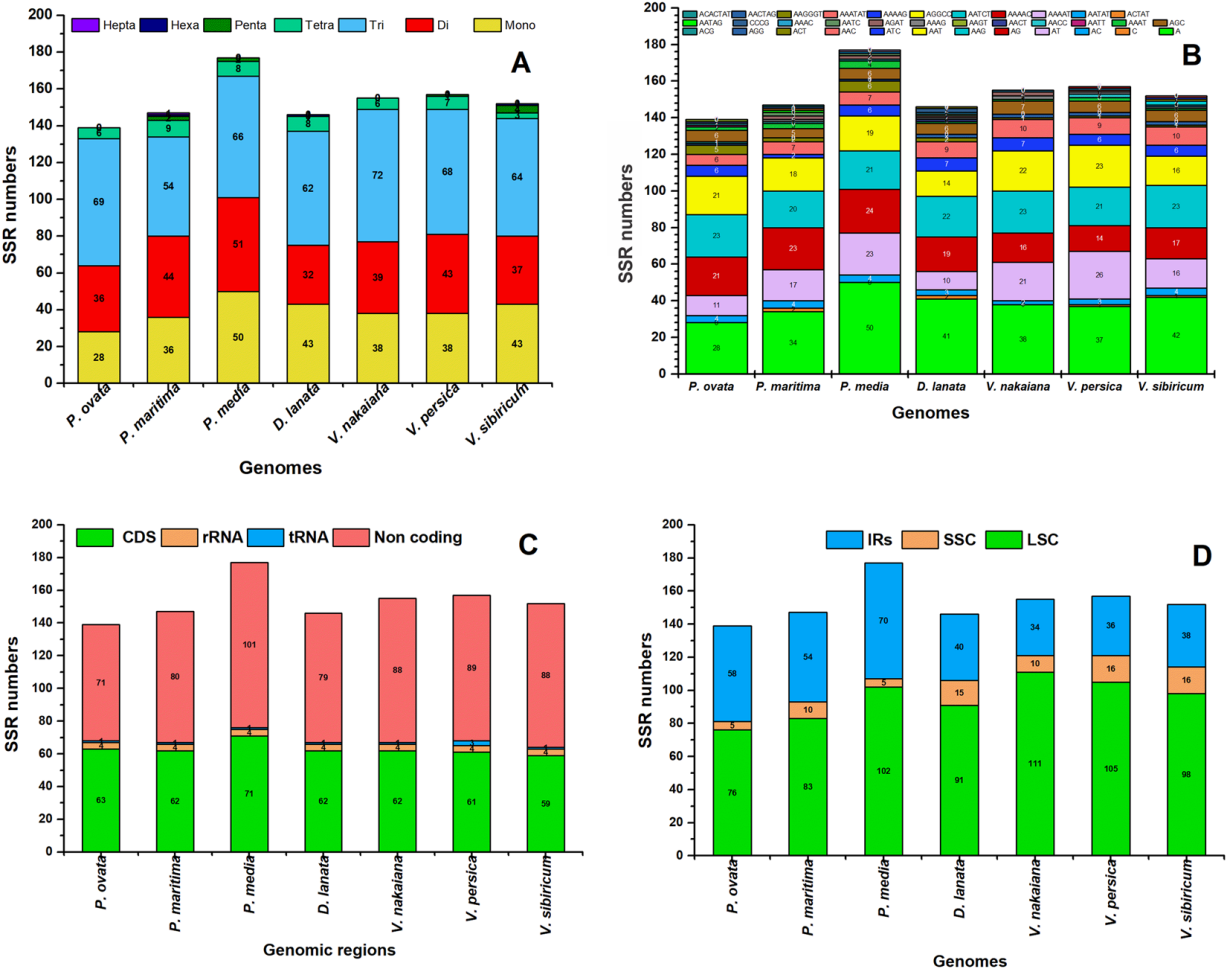
Analysis of simple sequence repeats (SSR) in the seven Plantaginaceae plastomes; (**A**) SSR numbers detected in the seven species; (**B**) Frequency of identified SSR motifs in different repeat class types; (**C**) Frequency of identified SSRs in coding, Non-coding, rRNA and tRNA regions; (**D**) Frequency of identified SSRs in LSC, SSC and IR regions.

In the present study, we determined SSRs in the plastome of *P. ovata* as well as in the plastomes of other seven other related species (Fig. 4). We examined the type, distribution, and occurrence of SSRs in plastomes. Based on SSR analysis, a total of 139 microsatellites were detected in *P. ovata* plastome. Among these, 71 SSRs were identified in non-coding regions, 63 in coding regions, four in rRNA-coding region, and 1 in tRNA-coding region. Similarly, in LSC, IRs, and SSC, 76, 58 and 5 SSRs were detected, respectively (Fig. 4). In *P. ovata* plastome, a majority of SSRs consisted of tri- (69, 49%) and di-nucleotide (36, 25.80%) repeats. This pattern is similar to that in the related genomes analysed in this study. We did not detect penta-, hexa-, and heptanucleotides in *P. ovata* plastome. However, in *P. maritima* and *P. media* plastomes, two penta-nucleotides were observed, whereas one hexa- and heptanucleotide were detected in *P. maritima* plastomes (Fig. 4; Table S5). In *P. ovata*, almost 100% of the mononucleotides contain an A motif, whereas a majority of di-nucleotide SSRs were A/G (21, 58.30%) and A/T (11, 30.50%), respectively. A similar pattern of SSR motif was observed in related plastomes (Fig. 4).

### Repeat sequence analysis

**Figure 5:**
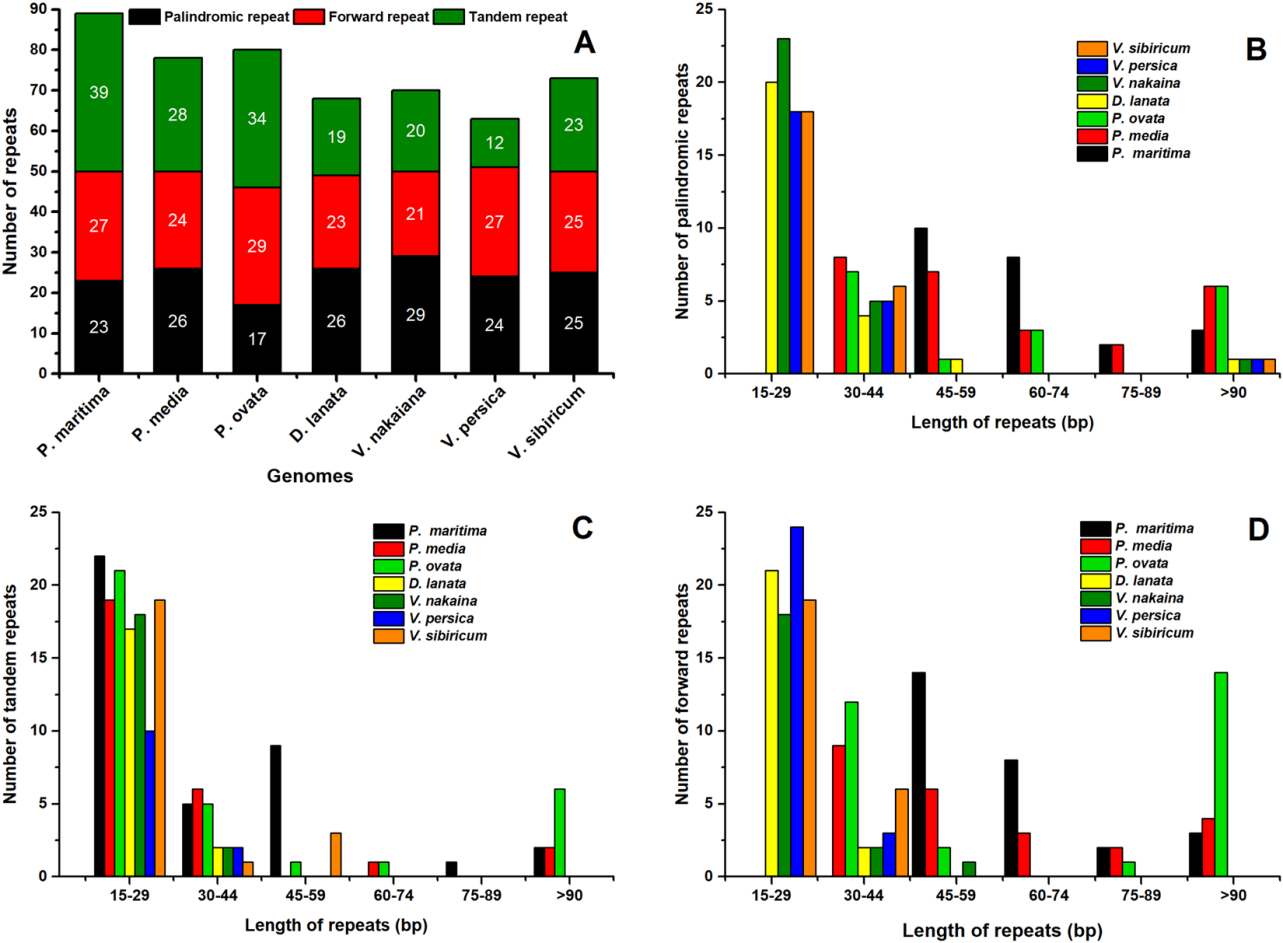
Analysis of repeated sequences in the seven *Plantaginaceae* plastomes. (**A**) Totals numbers of three repeat types; (**B**) Number of palindromic repeats by length; (**C**) Number of tandem repeats by length; (**D**) Number of forward repeats by length.

Repeat sequence analysis of *P. ovata* plastome with related species revealed the presence of 32 forward repeats, 34 tandem repeats, and 17 palindromic repeats (Fig. 5). Among these repeats, 7 of the palindromic repeats were 30–44 bp in length, while 6 repeats were >90 bp in length. Similarly, 12 and 14 forward repeats were 30–44 bp and >90 bp in length, respectively, whereas approximately 21 tandem repeats were identified to have a length of 15–29 bp (Fig. 5). Overall, 83 repeats were detected in *P*. *ovata* plastome, which is lower than those in *P. maritima* plastome (89) and higher than those in *V. persica* (63) and *D. lanata* (68) plastomes. Approximately 25% palindromic repeats, 17.60% forward repeats, and 26.60% tandem repeats were distributed in the protein-coding regions of *P. ovata* plastome (Tables S6, S7). Moreover, a higher number of palindromic repeats (29), forward repeats (32), and tandem repeats (39) were detected in *V. nakaiana, P. ovata* and *P. maritima* plastomes (Fig. 5).

### Contraction and expansion of IRs

IR regions are considered to be the most conserved regions in a chloroplast genome. The larger plastome sizes correlate with larger IR length; similar to the plastomes of two previously described *Plantago* species (*P. maritima* and *P. media*), *P. ovata* also contained IRs that significantly increased in size up to 37.40 kb as compared with IRs in the other related species (Table 1). Repeat expansion occurred in the SSC, resulting in the transfer of 9 former SSC genes (*rps15*, *ndhI*, *ndhD, psaC ndhA*, *ndhH*, *ndhG*, *ndhE*, and *ycf1*) into the IR regions (Fig. 1). Consistent with the observation in *P. media* and *P. maritima* plastomes, *P. ovata* also showed large-scale inversion of 13.80 kb within the expanded IR regions. For example, in *P. media* and *P. ovata* the breakpoint is inferred to be at *trnL-ndhB* and *trnN-trnR*, which are *ycf1-rps15* and *trnL-ndhB* in *P. maritima* (Fig. 1). The gene arrangement in the IR region of *P*. *ovata* is more similar to that of *P*. *media* than that of *P*. *maritima*, where sixteen protein-coding genes (*rpl2*, *rpl23, ycf15, rps12, rps7, ndhB*, *ycf1*, *rps15*, *ndhH*, *ndhA*, *ndhI*, *ndhG*, *ndhE*, *psaC* and *ndhD*) are duplicated. However, in *P*. *maritima* the duplicated protein coding genes are eleven and five former SSC genes transferred into IR region. Another noteworthy variation among *Plantago* plastomes IR regions was an additional small-scale inversion related with *ycf1* gene detected only in *P*. *maritima* plastome (Fig. S4).

**Figure 6:**
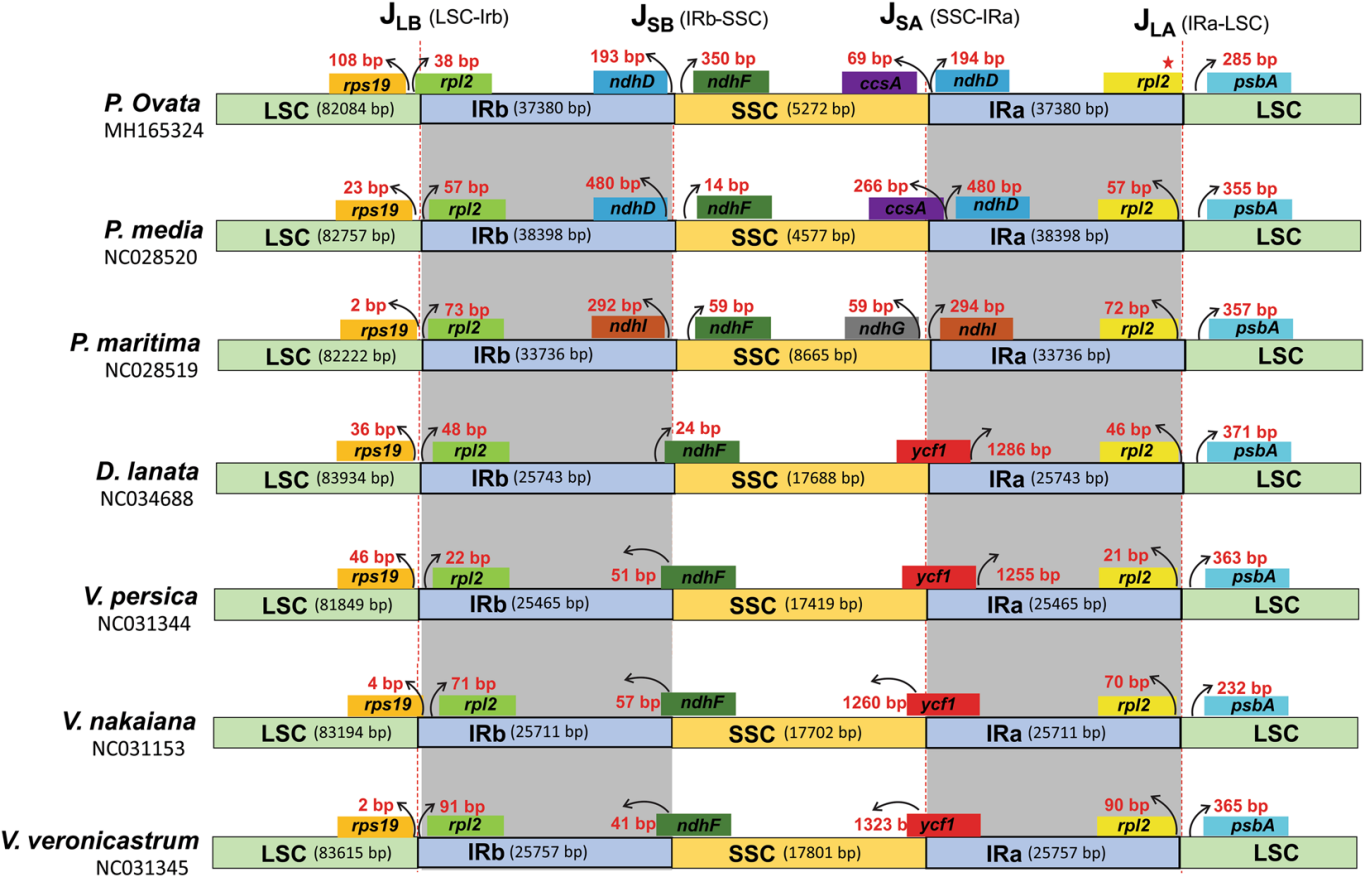
Comparison of border distance between adjacent genes and junctions of the LSC, SSC, and two IR regions among the plastome genomes of *P*. *ovata* and its relatives. Boxes above or below the main line indicate the adjacent border genes. The figure is not to scale with respect to sequence length and only shows relative changes at or near the IR/LSC or IR/SSC borders.

The IR/LSC and IR/SSC borders of *P. ovata* were compared with related plastomes (Fig. 6). The *rps19* gene was separated from the LSC/IRb region by 108 bp, and the *rpl2* gene extended to the LSC region and was duplicated in IR regions. Contrastingly, in other plastomes, *rpl2* did not extend up to the LSC region and was completely duplicated in IRs. Similarly, in *P. ovata*, owing to an extension of *rpl2* gene at LSC/IRb borders, IRa ended up with the truncated copy of *rpl2* gene. The position of *ndhF* gene in the SSC varied in these plastomes. In *P. ovata*, *P. media*, and *P. maritima, ndhF* gene was located 350, 14, and 59 bp away from IRb/SSC in the SSC, whereas in other related members of Plantaginaceae family, it extended up to the IRb region (Fig. 6). Furthermore, in *P. ovata*, the *ccsA* gene was 69 bp away from SSC/IRa border in the SSC, whereas *ndhD* gene was 194 bp away from this region and located in the IRa region. In case of *P. media, ccsA* extended to the IR region. Because the IR length in *P. maritima* is smaller than *P. ovata* and *P. media*, IR junction varied. Here, the *ndhI* was located 292 bp away from the IRb/SSC in the IRb region, whereas *ndhG* was 59 bp away from the SSC/IRa border in the SSC. In other *Plantaginaceae* members (*D. lanata*, *V. persica*, *V. nakaiana*, and *V. veronicstrum*), *ndhH* gene extended up to 24 bp, 51 bp, 57 bp, and 41 bp, respectively, into the IRb region at the IRb/SSC border. The *psbA* gene in all species was located in the LSC region and separated from the IRa/LSC border by 232–371 bp.

### Phylogenetic analysis and Divergence time of *P. ovata* with related species

**Figure 7:**
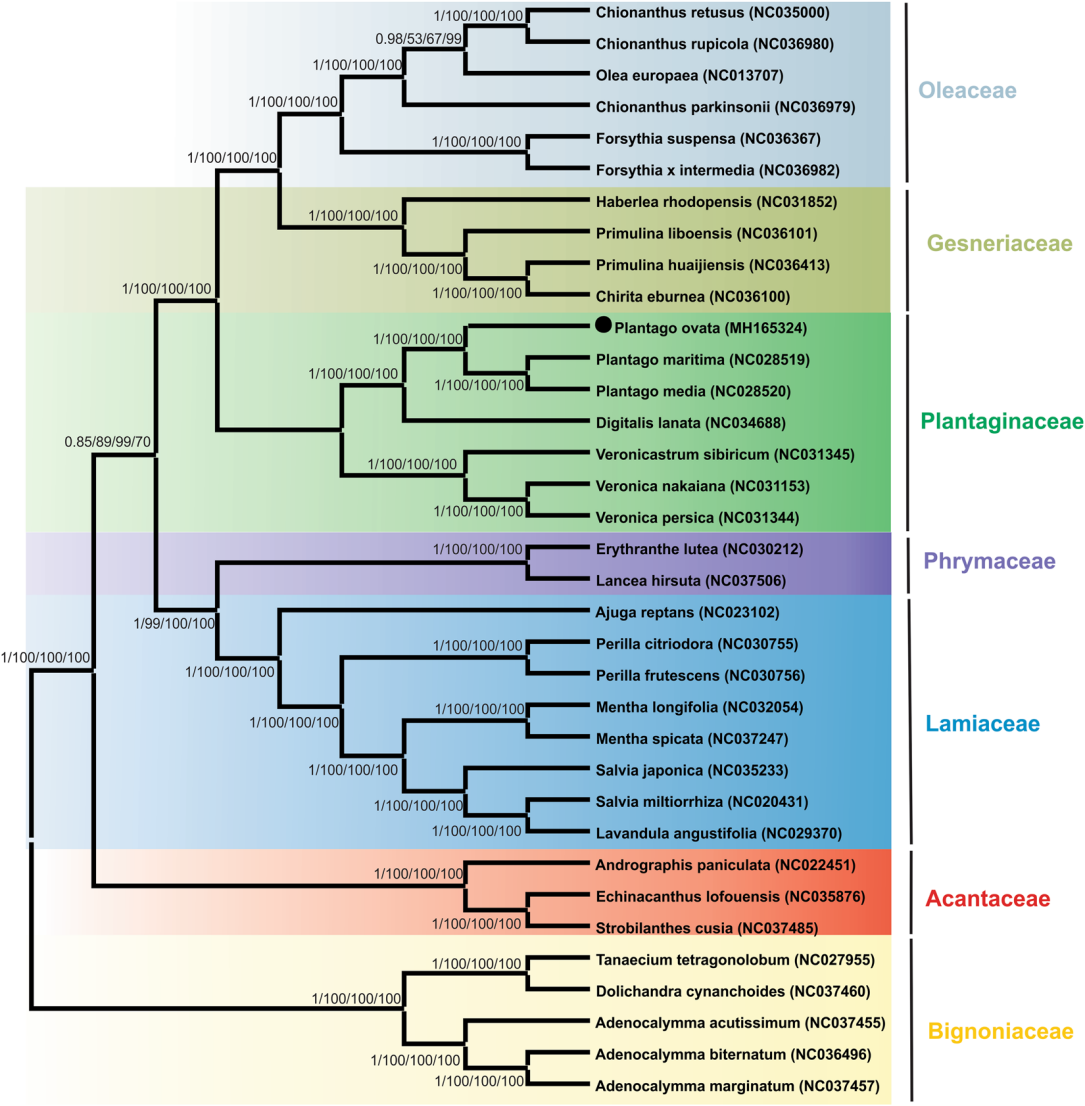
Phylogenetic trees were constructed for thirty-five species from eight families representing 22 genera using different methods, and tree is shown for the whole genome sequence data sets constructed by ML method. The whole genome sequence data set was used with four different methods, Bayesian inference (BI), maximum likelihood (ML), maximum persimony (MP) and neighbour-jouining (NJ). Numbers above the branches are the posterior probabilities of BI and bootstrap values of ML, MP and NJ respectively. Black dots represent the position for *P. ovata*.

Here, the phylogenetic position of *P. ovata* within the order Lamiales was established by multiple alignment analysis of the complete plastome, 72 shared genes, *matK* gene, and *rbcL* gene of Lamiales members representing 8 families and 22 genera (Figs. 7; S5). Phylogenetic analyses were performed using ML, NJ, MP, and BI methods. ML analysis revealed that 28 out of 30 nodes had a bootstrap value of ≥99%, while the remaining had a value of 100% (Figs. 7; S5). The phylogenetic trees constructed based on the sequences of the complete genome, 72 shared genes, *matK* gene, and *rbcL* gene of P. *ovata* formed a clade with *P. maritima* and *P. media* via bootstrap and BI support. In the analysed data sets, *D. lanata* was inferred to be closest to the *Plantago* than to *Veronica* species. Furthermore, *Gesneriaceae* and *Phrymaceae* were found to be closely related families on the basis of the sequences of their plastomes, 72 shared genes, *matK* gene, and *rbcL* gene (Figs. 7; S5). The divergence time was estimated with Baysean approach as implemented in BEAST showed that *P. ovata* has diverged from the common ancestor of *P. media* and *P. maritima* at 11.0 million years ago (Mya; 95% HPD, 10.06–12.25 Mya) (Fig. S6). Additionally, the tree implemented in BEAST resulted a congruent topology with those generated by ML, NJ, and MP.

## Discussion

In this study, we sequenced and annotated the complete plastome of *P. ovata* and compared it with the plastomes of related Plantaginaceae species. *Plantago ovata* plastome exhibited a typical quadripartite structure of 162,116 bp, correlated with other *Plantago* species (Table 1)^25^. Though gene content and organisation in *P. ovata* plastome found to be similar with other species in Plantaginaceae, however, the genome size and IRs of *P. ovata* was markedly different from *P. media* and *P. maritima*^25^. Similar to *P. media* and *P. maritima*, *P. ovata* plastome exhibited a higher level of sequence and structural divergence as well as showed some rearrangements in its plastome in comparison with those for typical angiosperms plastomes^25–27^. We observed a similar number of intron-containing protein-coding genes with 7 tRNA genes (with an exception of *ycf3* and *clpP* which contained two introns) in *P. media* plastome^25^. Conversely, in *P. maritima, V. persica, V. sibiricum, D. lanata*, and *V. nakaiana*, the number of intron-containing genes were 13, 14, 15, 15, and 15, respectively (Table 1)^25,28^. In synergy with other angiosperms, *rps12* gene was unevenly divided with its 5′ terminal exon located in the LSC region, whereas the 3′ terminal exon and intron were located in the IRb region and also duplicated in the IRa region^29,30^. The *trnK-UUU* had the longest intron (2,434 bp)^26,31^. According to a previous study, introns play a vital role in gene expression regulation; improve exogenous gene expression and transformational efficiency^32^. This intronic effect has been widely described in various plants^33–35^ and termed as intron-mediated enhancement^34–36^. In fact, because some introns are markedly efficient in triggering gene expression, they are regularly included to constructs for maintaining a high expression level^37^. Cysteine and leucine were the least and the most common amino acids in *P. ovata* plastome, respectively (Table S2). These findings were also supported by the analysis of previous plastomes of other related species belonging to Plantaginaceae family^25,28^. The presence of high AT content at the third codon position is consistent with that in the plastomes of various terrestrial plants^31,38–40^.

The present results revealed that *P. ovata* shared a high sequence similarity with all the analysed species. However, relatively lower identity was detected for these plastomes in various comparable genomic regions. Previously in angiosperm genomes, the SSC and LSC regions showed more divergence than the IR regions^25,27,29,41^. These results are in agreement with previous reports which showed a higher sequence divergence because of copy correction for gene conservation in IR regions^42^. The plastome of *P. ovata* showed a high divergence level in non-coding and intergenic spacer regions (*atpH-atpI, rpoC1-rpoC2, ycf1-rps15, accD-psaI, psaA-ycf3*, and *trnL-rrn5)* as opposed to the coding regions (Fig. 2)^26,39,43^. However, some divergent protein-coding genes (*accD, clpP, ndhA, ndhF, rpl16, petD, matK, rpl16, ycf2, ycf1*, and *rpl2*) were also detected in *P. ovata* plastome. This is in correlation with previously reported findings for the plastomes of angiosperms^31,43^. Manezes *et al*.^42^ concluded that divergent plastome genes are predominantly detected in the LSC regions, suggesting a more rapid evolution trend^44^. Our results are based on divergence time estimation that showed a rapid divergence trend in genus *Plantago* and revealed that *P. ovata* has diverged from *P. media* and *P. maritima* at 11.0 million years (Mya; 95% HPD, 10.06–12.25 Mya; Fig. S6). Similar results were reported by Iwanycki Ahlstrand *et al*.^45^ on the basis of various genes that subgenus *Plantago* diverged about 8.8 Mya (95% HPD, 17.4–2.5 Mya). These results are also consistent with the divergence time estimation of family Plantaginaceae where its crown age was estimated 31.42 Mya^46^. The highest average pairwise distances were detected in *clpP* and *accD* genes of the *P. ovata* plastome (Fig. 3). This divergence was observed in *accD* gene because the plastomes of three *Plantago* species had different gene lengths. Previous studies have also observed varied lengths of *accD* gene within an angiosperm plastome^26,47^. In legume, this variation in length was partially explained by the presence of various tandemly repeated sequences^47^. Knockout experiments showed important functions of *accD* gene (encodes acetyl-CoA)^48^. However, in various relatively distant lineages (for example, *Fabaceae*, *Poaceae*, *Lamiaceae*, *Geraniaceae*, *Campanulaceae*, and *Ericaceae*), the *accD* gene was identified as pseudogene and completely absent which shows that the deletion or pseudogenisation even of *accD* occur independently^26,49–51^. The second most divergent gene, *clpP*, showed highest divergence with *P. maritima*, which can be a result of introns loss in *P. maritima* genome as reported previously by Zhu *et al*.^25^.

SSRs, on the other hand, are valuable markers to detect a high-degree variation with the same species and have been used to investigate polymorphisms and population genetics^52^. We have detected 139 microsatellites in *P. ovata* plastome and about 71 were found in non-coding regions. This is consistent with angiosperm plastomes in which the highest number of SSRs are detected mostly in non-coding regions^29,39,53^. Approximately, 147, 177, 146, 155, 157, and 152 SSRs were detected in *P. maritima*, *P. media, D. lanata, V. persica, V. nakaiana*, and *V. sibiricum* plastomes, respectively (Fig. 4). Tri- and dinucleotide repeats were predominantly present in *P. ovata*. This pattern was also observed in a related genome described previously^28^. An angiosperm plastome mostly contains mononucleated A motif, whereas most di-nucleotides contain A/G motif^40,53,54^. Mononucleotide, pentanucleotide, and hexanucleotide repeats contain “A” or “T” bases at higher frequencies, suggesting a biased base composition with an overall A-T richness in the plastomes^55,56^. Similar previous reports have revealed an uneven distribution of SSRs in *Plantago* species, owing to which it may be possible to procure more valuable information regarding effective molecular markers for intraspecific polymorphism^57,58^. We determined 32 forward, 34 tandem, and 17 palindromic repeats in *P. ovata* plastome. Repeat sequences have been reported in the plastomes of various plant lineages^28,44,59^. These types of sequences are very important and used extensively for a range of evolutionary and population genetic studies^60,61^. Additionally, repeat sequences play a central role in plastome rearrangement^62,63^. The length of palindromic and direct repeats in *P. ovata* were considerably long, ranging from 340 bp to 532 bp (Table S7); similar results have been previously reported for the plastomes of *Poaceae, Fabaceae*, *Clematis*, and *Ranunculus* species^39,44,64^. As reported previously, genome rearrangement and sequence diversity occur owing to an incorrect recombination of these repeat sequences and slipped strand mispairing^65,66^. Furthermore, the occurrence of these repeats indicate that the region is a critical hotspot for the reconfiguration of plastome genome^66^. Additionally, these repeats are an informative source for developing genetic markers for *Plantago* species, which can be further applied in phylogenetic and population studies^62^.

Regarding IR regions, contraction and expansion at borders are the key motives underlying size variations in plastome genomes, and thereby play an important role in evolution^48,67,68^. Compared with a typical angiosperm genome, in *P. ovata* plastome, the IR region significantly increased in size up to 37.4 kb, which indicates the transfer of nine SSC genes to the IR regions. Similar results were observed in the previously reported plastomes of *P. media* and *P. maritima*, where 9 and 5 gene transfers occurred into the IR region, respectively^25^. An inversion of 13.8 kb was detected in *P. ovata* plastome, which was 21 kb and 14 kb in *P. maritima* and *P. media* plastomes, respectively (Fig. S4). The increase in total genome size can be explained by an increase in the length of IRs, as reported previously in various plastomes^25,41,69^. Previously, larger repeats ranging from >25 to 30 kb in size were also reported in angiosperms, and small size-related changes of a few hundred base pairs commonly occur in IR^27,70^. However, some larger and rare changes in the size of IR regions, ranging from 43,864 bp in *Buchnera* to 63,240 bp in *S. forbessii* have been reported^71^. The largest known repeat of 75,741 bp was reported in *Geranium hortorum* by Chumley, *et al*.^72^. During land plant evolution, there have been multiple instances of IR expansion and contraction which caused the displacement of an entire gene from the SC regions into the IR regions or from the IR to SSC regions^25,71^. In *P. ovata* plastome, the terminal IR gene adjacent to the SSC is highly conserve as reported previously^25,71^. In most species, *trnN-GUU* is a full-length IR gene present at the IR/SSC boundary, which provides a strong evidence for ancestral IR/SSC endpoint that has been conserved in most lineages^25^. However, similar to *P. media* and *P. maritima*, some degree of extension into the SSC was observed in *P. ovata* plastome which to some extent occurred in other angiosperms^25,73^. Similar to the other two *Plantago* species, in *P. ovata* plastome, IR expansion has distinct features as opposed to an enlarged IR lineage such as *Berberis*, *Trochodenfron*, and *N. acuminate*^25^. These features include an extensive genomic rearrangement, which suggest that a different mechanism of IR expansion may be involved. A similar mechanism was observed in *Pelargonium* IR expansion, which suggested a model involving multiple inversion promoted by these enlarged repeats^72^. In *Plantago* plastome, these repeats indicate that they may be involved in inversion events as reported previously^25^. Moreover, a detailed comparison between IR/LSC and IR/SSC border of *P. ovata*, which was subsequently compared with the borders in other related plastomes (Fig. 6). We carefully analysed and compared the exact IR border position and its adjacent genes among the plastomes of other Plantaginaceae species. The study revealed that the LSC/IRb was located between the *rps19* and *rpl2* genes in all Plantaginaceae plastomes. However, the *rpl2* gene extended into the IRb regions ranging from 22 bp (*V. persica*) to 91 bp (*V. veronicastrum*) in all the analysed plastomes, except *P. ovata*, which had a 38-bp extension into the LSC regions. This distance was 57 bp and 73 bp away from the LSC/IRb border in *P. media* and *P. maritima*, respectively. Due to the smaller IR length of *P. maritima* among *Plantago* species, IR junctions vary. Previous studies have revealed that there is an expansion of the IR and LSC regions in angiosperm plastomes during evolution^25,70,72^.

The *Plantago* genus comprises approximately of 200 species^17^, most of which are mainly cross-pollinated in nature^74^. Some of these species are medicinally important and various wild species reportedly possess important genes that play a vital role in isabgol production when introgressed to cultivated species^18^. Continued studies have extended our knowledge to distinguish and understand the genomic structure of and phylogenetic relationships among *Plantago* species^23,74,75^. Taxonomy and phylogeny of the *Plantago* genus within Lamiales have been widely examined at the genus level^20,22^. Previous evolutionary relationships among different *Plantago* species were estimated by analysing nuclear polymorphisms, random amplified polymorphic DNA (RAPD) profiles, chloroplast DNA restriction fragment-length polymorphisms (RFLP), SSR region, and ITS region as well as the sequences of 5 S rRNA genes, rps14 gene, and plastid *trnL-F*^20,22,75,76^. However, complete genome sequencing provides more detailed insight^31,77,78^. In this regard, the complete genome sequence of *P. ovata* has been overlooked; therefore, the current dataset will provide more detailed insights into the role of various genes for understanding the plant’s life in a better manner. The plastome genomes have shown considerable applicability in phylogenetic studies and molecular and evolution systematics. During the recent years, various analyses have been conducted at deep nodes to answer phylogenetic questions based on the entire plastome genome and compared with multiple protein-coding genes^79,80^; this facilitates better understanding of complex evolutionary relationships among angiosperms^81^. Therefore, in this study, the phylogenetic position of *P. ovata* within *Plantago* and Lamiales was established by utilizing the complete plastome, 72 shared genes, *matK* gene, and *rbcL* gene among the members of 8 families representing 22 genera (Figs. 7 and S4). Phylogenetic analysis using BI and ML methods were performed. The results revealed that complete genome sequences (Fig. 7), 72 shared genes, *matK* gene, and *rbcL* gene (Fig. 4) from all the analysed species generated a phylogenetic tree with the same topology. In these phylogenetic trees (Figs. 7 and S4) constructed by employing ML, MP, NJ, and BI methods, *P*. *ovata* formed a single clade with *P. maritima* and *P. media* with high bootstrap (100%) and BI support. Moreover, the tree topology enabled inference of the relationship based on the phylogenetic studies conducted by Nina (2002)^22^ and Zhu (2015)^25^. The position of *P. ovata* within Lamiales confirms the previously published phylogeny described by Schäferhoff, *et al*.^82^. It was stated that Plantaginaceae is more closer to Gesneriaceae and Scrophulariaceae than Lamiaceae^82^. However, the chloroplast (cp) genome from Scrophulariaceae was not included owing to missing data in the NCBI database. Therefore, the present phylogeny revealed that Plantaginaceae is closer to Gesneriaceae and Phrymaceae on the basis of a complete plastome data set (Fig. 7). Similar results were observed on the basis of *rbcL* and *matK* genes (Figure S4), and were also consistent with the findings reported by Bastian *et al*.^82^. The results obtained here will help to recognise the evolutionary history of the Plantaginaceae. Furthermore, these results suggest that Plantaginaceae germplasm-related genetic resources are valuable and informative material for species identification, taxonomy elucidation, and phylogenetic inference of Plantaginaceae species. Moreover, phylogenetic inferences within Lamiales and Plantaginaceae could be improved if plastid genomes are made available, potentially providing dozens of valuable molecular markers for further research.

## Conclusion

The current findings reveal detailed insights of complete plastome genome of *P*. *ovata* for the first time through sequencing on Illumina HiSeq-2000 platform. The structure and gene contents of *P*. *ovata* plastome was found in synergy with related species in Plantaginaceae. Contrarily, the genome size and IRs of *P*. *ovata* were different from *P*. *media* and *P*. *maritima*. Through detailed bioinformatic analysis and comparative assessments, we retrieved essential genetic features such as repetitive sequences, SSRs, codon usage, IR contraction and expansion, Ka/Ks ratio, sequence divergence, divergence time and phylogenomic placement. More interestingly, we noticed the IR regions were found significantly increased in size (up to 37.4 kb), indicating transfer of nine SSC genes to the IR regions. Whilst, an inversion was comparatively similar between *P*. *ovata* and *P*. *media* but higher in *P. maritima*. The present phylogeny revealed that Plantaginaceae is closer to Gesneriaceae and Phrymaceae based on complete plastome datasets. The divergence time estimates showed that *P*. *ovata* diverge from common ancestor *P. media* and *P. maritima* around 11.18 million years ago (Mya). Current plastome genomic dataset and the detailed analysis of *P. ovata* and related species and their comparative analysis provides a powerful genetic resource for the future molecular phylogeny, evolution, population genetics and biological functions of genus *Plantago*.

## Materials and Methods

### Plastome sequencing and assembly

The fresh leaves of *P. ovata* were collected from District Dir Pakistan, and the collected sampled were immediately placed in liquid nitrogen and subsequently stored at −80 °C until DNA extraction. Young leaves were used to extract plastome DNA by following the protocol described by Shi *et al*.^83^ with numerous modifications, as described by Al-Dous *et al*.^84^. The Illumina HiSeq-2000 platform (San Diego, CA, USA) at Macrogen (Seoul, Korea) was used to sequence the resultant DNA. A total of 24,100,324 raw reads were generated for *P. ovata*, and CLC Genomics Workbench v7.0 (CLC Bio, Aarhus, Denmark) was used to trim and filter reads for the *de novo* genome assembly. Trimmomatic 0.36 was used for filtering the reads and trailing and leading nucleotide with a Phred score of <20 or when the Phred score dropped below 20 on implementing a 4-bp sliding-window approach. Similarly, reads of <50 bp were discarded after quality filtering and adaptor trimming. The first assembly was formed using SPADES v3.9.0, with an additional switchover to SOAPdenovo v2.04.

The resulting contigs were compared against the chloroplast genomes of *P. maritima* and *P. media* using BLASTN with an E-value cut-off of 1e-5. The regions which were uncertain in these genomes, such as IR junction’s region, were selected from the already published genome mentioned above to adjust the sequence length using the iteration method and by employing the Geneious v11.1.2 software^85^. Primers were procured from Macrogen Inc., South Korea to execute PCR amplification and Sanger sequencing to fill the gaps in previously reported data^29^. After incorporating the results of Sanger sequencing, the entire plastome was used as reference, and the initial short reads were remapped to refine the assembly and to get maximum coverage. From all the available data, only high quality reads were mapped back by using Bowtie2 in Geneious 11.1.2^85^.

### Genome annotation

The software, Dual organellar Genome Annotator (DOGMA)^86^, was used for annotating the *P*. *ovata* plastome genome. BLASTX and BLASTN were utilised to determine ribosomal RNAs, transfer RNAs, and the positions of coding genes. The tRNA genes were annotated by employing tRNAscan-SE, version 1.21, software under default settings^87^. Boundaries of genes, coding regions, exons, and introns were confirmed by using BLAST versus reference sequences. Furthermore, for manual adjustment, the start and stop codons and intron boundaries were manually adjusted with the help of the reference genome using Geneious (v. 11.1.2)^85^. Furthermore, for structural description, OGDRAW was used^88^. MEGA 6 software^89^ was used to determine the relative synonymous codon usage by avoiding the effect of amino acid composition. Finally, the divergence of the new *P. ovata* plastome from related species of family Plantaginaceae was assessed using mVISTA^90^ in Shuffle-LAGAN mode and by employing the new *P. ovata* genome as reference.

### Characterisation of repetitive sequences and SSRs

REPuter was used to determine the repetitive sequences (direct, reverse, and palindromic repeats within these plastomes^91^. For repeat identification via REPuter, the following settings were used: (1) a minimum repeat size of 30 bp, (2) ≥90% sequence identity, and (3) Hamming distance of 1. Tandem Repeats Finder version 4.07 b was used to find tandem repeats by using default settings^92^. Similarly, for finding SSRs, the search parameters were set to ≥3 repeat units for pentanucleotide and hexanucleotide repeats, ≥4 repeat units for trinucleotide and tetranucleotide repeats, ≥8 repeat units for dinucleotide repeats, and ≥10 repeat units for mononucleotide repeats by employing Phobos version 3.3.12^93^.

### Sequence divergence, phylogenetic analyses and divergence time

Entire plastomes genomes and an isolated partition comprising 72 shared genes were used to examine the average pairwise sequence divergence for six species (*P. media, P. maritima, V. nakaiana*, *V. presica, V. veronicastrum*, and *D. lanata*) from the Plantaginaceae family. Ambiguous and missing gene annotations were checked by conducting comparative sequence analysis after assembling a multiple sequence alignment and comparing the gene order. These datasets were aligned using MAFFT v7.222^94^ under default parameters, and Kimura’s two-parameter (K2P) model was used to calculate pairwise sequence divergence^95^. Similarly, DnaSP v5.10.01^96^ was used to indemnify InDel polymorphisms among these plastomes, and for identifying single-nucleotide polymorphisms, a custom Python script (https://www.biostars.org/p/119214/) was employed. To resolve the phylogenetic position of *P. ovata* within the family Plantaginaceae and order Lamiales, 34 published plastome sequences of Lamiales species were downloaded from the NCBI database for phylogenetic analysis. First, on the basis of a conserved structure and gene order, a multiple alignment of the complete plastomes was created^97^, and the following four methods were applied to construct phylogenetic trees by employing the settings described previously: Bayesian inference (BI) was employed in MrBayes 3.1.2^98^; maximum likelihood (ML) and neighbour-joining (NJ) were as implemented in MEGA 6^89^; and maximum parsimony (MP) by using PAUP^41,99^. The best substitution model GTR + G was tested by jModelTest version v2.1.02^100^ according to the Akaike information criterion (AIC) for Bayesian posterior probabilities (PP) in BI analyses. The Markov Chain Monte Carlo (MCMC) method was run using four incrementally heated chains across 1,000,000 generations, starting from random trees and sampling 1 out of every 100 generations. To estimate the posterior probabilities, the values of first 30% of trees were discarded as burn-in. Maximum parsimony run was based on a heuristic search with 1000 random addition of sequence replicates with the tree-bisection-reconnection (TBR) branch-swapping tree search criterion. Similarly, the parameters for ML analysis were optimised using a BIONJ tree^101^ as the starting tree with 1000 bootstrap replicates by employing the Kimura 2-parameter model with invariant sites and gamma-distributed rate heterogeneity.

In the second, third, and fourth tiers of phylogenies, a set of 72 shared genes, *matK* gene, and *rbcL* gene from the plastome genomes of 34 Lamiales species were aligned using MAFFT version 7.222^94^ under default parameters and by making various manual adjustments to preserve and improve reading frames. The above two aforementioned phylogenetic inference models (ML and BI) were employed to construct trees using 72 concatenated genes, *matK* gene, and *rbcL* gene as mentioned above and suggested by Asaf *et al*.^29^.

To determine the divergence time of *Plantago* with those of other 34 species, we used the concatenated data matrix. Briefly general time reversible (GTR + G) substitution model was used with four rate categories, and a Yule tree speciation model with lognormal relaxed clock model in BEAST^102^ with a substitution rate prior. We used an average substitution rate of 3.0 × 10^−9^ substitutions per site per year (s/s/y) and a fossil-based method to calibrate the molecular divergence. To root the calibration time, we included five outgroups species as *Dolichandra cyanachoides*, *Tanaecium tetragonolobum*, *Adenocalymma acutissimum*, *Adenocalymma biternatum* and *Adenocalymma marginatum from* family Bignoniaceae. We selected these outgroups as all these species are closely related to our study model species and have fossil records older than genus *Plantago*^103^. The fossil records were employed through Log-normal distributed priors root set to the node ages of family Bignoniaceae 49.5 Mya (Offset = 45.0, Mean = 1.5 and SD = 0.5)^104–106^. The dating analyses involved 3 independent MCMC runs of 15 million generations. LOGCOMBINER was used to combine the tree files from all the three runs. Convergence and effective sample sizes were assessed in TRACER 1.5^107^. From each analysis we removed 25% of trees as burn-in. Lastly, the tree was calculated using TREEANNOTATOR and tree with 95% highest posterior density (HPD) was visualized in FIGTREE1.4.

## Supplementary information

Suplemenetry information.

Supplementary data set.
