# Supplementary material for: Expanded inverted repeat region with large scale inversion in the first complete plastid genome sequence of Plantago ovata
Source: Sci Rep. 2020 Mar 3;10:3881. doi: 10.1038/s41598-020-60803-y (PMC7054531; doi:10.1038/s41598-020-60803-y)
Supplement: Supplementary file 1 — Suplemenetry information. [file 41598_2020_60803_MOESM1_ESM.docx]

**Expanded inverted repeat region with large scale inversion in the first complete plastid genome sequence of *Plantago ovata***

Sajjad Asaf^1^, Abdul Latif Khan^1^*, Lubna^2^, Adil khan^1^, Arif Khan^1^, Gulzar Khan^3^, In-Jung Lee^4^*, Ahmed Al-Harrasi^1^*,

^1^ Natural and Medical Sciences Research Center, University of Nizwa, Nizwa, Oman

^2^Department of Botany, Garden Campus, Abdul Wali Khan University Mardan, Pakistan

^3^Institute for Biology and Environmental Sciences, Carl von Ossietzky University, Oldenburg, Oldenburg, Germany

^4^School of Applied Biosciences, Kyungpook National University, Daegu 41566, Republic of Korea

**Corresponding Authors:**

Abdul Latif Khan ([latifepm78@yahoo.co.uk](mailto:latifepm78@yahoo.co.uk))

In-Jung Lee (ijlee@knu.ac.kr)

Ahmed Al-Harrasi ([aharrasi@unizwa.edu.om)](mailto:aharrasi@unizwa.edu.om))

**Figure S1**. Ka/ks values of the *accD*, *clpP* and complete plastome of the chloroplast genomes of the *P. ovata* with related species.

**Figure S2**. Alignment of *accD* gene nucleotide sequences among 7 *Plantaganaceae* species plastomes.

**Figure S3**. Alignment of *clpP* gene nucleotide sequences among 7 *Plantaganaceae* species plastomes.

**Figure S4**. Alignment visualization of the IR regions among *P. ovata*, *P. media* and *P. maritima* plastome genomes. **(A)** IR regions are aligned and drawn with the OGDRAW online tool ((http://ogdraw.mpimp-golm.mpg.de) and large inversion regions are indicated in blue color. Green color shows additional inversion found only in *P. maritima* IR region. **(B)** MAUVE alignment of the IR regions of *Plantago* plastome sequences. *P. ovata* IR region was used as a reference.

**Figure S5.** Phylogenetic trees were constructed for thirty-five species from eight families representing 22 genera using different methods, and tree is shown for the 72 protein coding genes **(A)**, *matK* (**B)** and *rbcL* **(C)** data sets. These sequences data sets were used with two different methods, Bayesian inference (BI) and maximum likelihood (ML). Numbers above the branches are the posterior probabilities of BI and bootstrap values of ML respectively. Black dots represent the position for *P. ovata.*

**Figure S6**. Bayesian chronogram of divergence time for *Plantago* species as employed in BEAST 1.8.5 [^106^](#_ENREF_109) rooted with family Bignoniaceae (*Dolichandra cyanachoides*, *Tanaecium tetragonolobum*, *Adenocalymma acutissimum*, *Adenocalymma biternatum* and *Adenocalymma marginatum*). General time reversible (GTR + G) substitution model was used with four rate categories, and a Yule tree speciation model with lognormal relaxed clock model in BEAST. The number near the nodes represent divergence time in million years, blue bar indicating 95% highest posterior densities.

**Table S1.** Base compositions in the *P. ovata* plastome with related species.

**Table S2**. The codon–anticodon recognition pattern and codon usage for the *P. ovata* chloroplast genome**.**

**Table S3.** Average pairwise sequence distance of *P. ovata* with related species plastomes

**Table S4.** Indel and SNP analysis of *P. ovata* plastome with related species.

**Table S5.** Simple sequence repeats (SSRs) in *P. ovata* plastome genome

**Table S6.** Tandem repeats in *P. ovata* plastome.

**Table S7.** Forward and palindromic repeats in *P. ovata* plastome.

| **Table S1.** Base compositions in the *P*. *ovata* plastome with related species. |
| --- |

This table is provided in the form of a single Supplementary Dataset file.

**Table S2**. The codon–anticodon recognition pattern and codon usage for the *P. ovata* chloroplast genome**.**

| **Amino acid** | **Codon** | **No** | **RSCU** | **tRNA** | **Amino acid** | **Codon** | **No** | **RSCU** | **tRNA** |
| --- | --- | --- | --- | --- | --- | --- | --- | --- | --- |
| Phe | UUU | 2436 | 1.23 |  | Tyr | UAC | 696 | 0.70 | *trnY-GUA* |
| Phe | UUC | 1519 | 0.77 | *trnF-GAA* | Tyr | UAU | 1297 | 1.30 |  |
| Leu | UUA | 1075 | 1.19 | *trnL-UAA* | Stop | UAA | 1152 | 1.15 |  |
| Leu | UUG | 1144 | 1.27 | *trnL-CAA* | Stop | UGA | 1010 | 1.01 |  |
| Leu | CUU | 1149 | 1.28 |  | Stop | UAG | 843 | 0.84 |  |
| Leu | CUC | 657 | 0.55 |  | Cyc | UGC | 484 | 0.84 | *trnC-GCA* |
| Leu | CUA | 882 | 0.98 | *trnL-UAG* | Cyc | UGU | 671 | 1.16 |  |
| Leu | CUG | 496 | 0.55 |  | Trp | UGG | 781 | 1.00 | *trnW-CCA* |
| Ile | AUU | 1784 | 1.23 |  | His | CAU | 819 | 1.34 |  |
| Ile | AUC | 1119 | 0.77 | *trnI-GAU* | His | CAC | 402 | 0.66 | *trnH-GUG* |
| Ile | AUA | 1443 | 1.00 | *trnI-CAU* | Gln | CAA | 1073 | 1.39 | *trnQ-UUG* |
| Met | AUG | 875 | 1.00 | *trn(f)M-CAU* | Gln | CAG | 468 | 0.61 |  |
| Val | GUU | 849 | 1.34 |  | Asn | AAU | 795 | 1.36 |  |
| Val | GUC | 495 | 0.78 | *trnV-GAC* | Asn | AAC | 845 | 0.64 | *trnN-GUU* |
| Val | GUA | 774 | 1.23 | *trnV-UAC* | Lys | AAA | 2266 | 1.35 | *trnK-UUU* |
| Val | GUG | 408 | 0.65 |  | Lys | AAG | 1099 | 0.65 |  |
| Ser | UCU | 1189 | 1.45 |  | Asp | GAU | 1101 | 1.45 |  |
| Ser | UCC | 889 | 1.08 | *trnS-GGA* | Asp | GAC | 414 | 0.55 | *trnD-GUC* |
| Ser | UCA | 982 | 1.20 | *trnS-UGA* | Glu | GAA | 1481 | 1.39 | *trnE-UUC* |
| Ser | UCG | 675 | 0.82 |  | Glu | GAG | 657 | 0.61 |  |
| Pro | CCU | 708 | 1.06 |  | Arg | CGU | 486 | 0.84 | *trnR-ACG* |
| Pro | CCC | 664 | 0.99 | *trnP-GGG* | Arg | CGC | 269 | 0.47 |  |
| Pro | CCA | 817 | 1.22 | *trnP-UGG* | Arg | CGA | 604 | 1.05 |  |
| Pro | CCG | 486 | 0.73 |  | Arg | CGG | 381 | 0.66 |  |
| Thr | ACU | 694 | 1.16 |  | Ser | AGU | 687 | 0.84 |  |
| Thr | ACC | 628 | 1.05 |  | Ser | AGC | 504 | 0.61 | *trnS-GCU* |
| Thr | ACA | 677 | 1.13 | *trnT-UGU* | Arg | AGA | 1087 | 1.88 | *trnR-UCU* |
| Thr | ACG | 401 | 0.67 |  | Arg | AGG | 636 | 1.10 |  |
| Ala | GCU | 568 | 1.32 |  | Gly | GGU | 621 | 1.02 |  |
| Ala | GCC | 392 | 0.91 |  | Gly | GGC | 356 | 0.59 | *trnG-GCC* |
| Ala | GCA | 487 | 1.14 | *trnA-UGC* | Gly | GGA | 840 | 1.38 | *trnG-UCC* |
| Ala | GCG | 269 | 0.63 |  | Gly | GGG | 613 | 1.01 |  |

| **Table S3. Average pairwise sequence distance of *P*. *ovata* with related species plastomes** |
| --- |

This table is provided in the form of a single Supplementary Dataset file.

**Table S4. Indel and SNP analysis of P. ovata plastome with related species.**

This table is provided in the form of a single Supplementary Dataset file.

**Table S5. Simple sequence repeats (SSRs) in P. ovata plastome genome**

| **Unit** | **Length** | **NO** | **SSR start** |
| --- | --- | --- | --- |
| **A** | 14 | 2 | 73036, 78422 |
|  | 13 | 1 | 26585 |
|  | 12 | 3 | 1532, 91487, 152704 |
|  | 11 | 7 | 7346, 57381, 75775, 76027, 81996, 106221, 137971 |
|  | 10 | 15 | 8106, 15591, 17627, 29045, 59117, 81574, 105759, 107977, 108504, 109687, 120517, 134506, 135689, 136216, 138434 |
| **AC** | 9 | 1 | 11958 |
|  | 8 | 3 | 3608, 115507, 128688 |
| **AT** | 10 | 3 | 7707, 19020, 35245 |
|  | 9 | 4 | 7698, 34261, 53061, 63631 |
|  | 8 | 4 | 35263, 55708, 72353, 120426 |
| **AG** | 9 | 4 | 7430, 18609, 34413, 40045 |
|  | 8 | 17 | 53746, 59032, 67625, 68192, 69306, 84276, 84834, 84951, 84963, 93604, 104239, 139956, 150591, 159232, 159244, 159361, 159919 |
| **AAG** | 11 | 5 | 18179, 21479, 71222, 119342, 124850 |
|  | 10 | 3 | 82102, 129357, 162091 |
|  | 9 | 13 | 36044, 76122, 80469, 89378, 103943, 106107, 107606, 119328, 121955, 124866, 138087, 140251, 154816 |
| **AAT** | 16 | 1 | 53237 |
|  | 12 | 3 | 45159, 107477, 136714 |
|  | 11 | 3 | 34248, 57779, 73218 |
|  | 10 | 4 | 27843, 78444, 111435, 132758 |
|  | 9 | 10 | 6295, 14668, 26942, 34731, 42468, 52628, 56056, 81582, 120969, 121466 |
| **ATC** | 10 | 1 | 37563 |
|  | 9 | 5 | 19523, 34771, 81148, 88160, 156034 |
| **AAC** | 9 | 6 | 13827, 48280, 65701, 70589, 105816, 138378 |
| **ACT** | 9 |  | 19927, 20134, 44553, 88440, 155754 |
| **AGC** | 9 | 6 | 77076, 81407, 95163, 113545, 130649, 149031 |
| **AAG** | 10 | 1 | 114836, 136588 |
|  | 9 | 1 |  |
| **ACG** | 22 | 1 | 69378 |
| **AGG** | 9 | 1 | 17815 |
| **AAAT** | 12 | 2 | 119068, 125123 |
| **AACT** | 13 | 1 | 14544 |
| **AATT** | 13 | 1 | 61276 |
| **AAGT** | 12 | 1 | 41871 |
| **AACC** | 12 | 1 | 70851 |

**Table S6. Tandem repeats in *P. ovata* plastome.**

| **Serial No** | **Indices** | **Period Size** | **Copy Number** | **Consensus Size** | **A** | **C** | **G** | **T** | **Location** |
| --- | --- | --- | --- | --- | --- | --- | --- | --- | --- |
| 1 | 11690 -11715 | 13 | 2.0 | 13 | 38 | 23 | 0 | 38 | CSD(*ndhE*) |
| 2 | 19787-20170 | 207 | 1.9 | 206 | 24 | 20 | 22 | 33 | IGS(*rpoC2*-*rpoC1*) |
| 3 | 26203-26227 | 13 | 1.9 | 13 | 32 | 12 | 0 | 56 | IGS(*rpoB-trnC-GCA*) |
| 4 | 26765-26802 | 19 | 2.0 | 19 | 42 | 5 | 5 | 47 | IGS(*trnC-GCA-petN*) |
| 5 | 26931-26960 | 14 | 2.1 | 14 | 66 | 0 | 0 | 33 | IGS(*trnC-GCA-petN*) |
| 6 | 28433-28487 | 18 | 3.1 | 18 | 49 | 16 | 5 | 29 | IGS(*petN-psbM*) |
| 7 | 30917-30950 | 17 | 2.0 | 17 | 14 | 14 | 5 | 64 | IGS(*trnT-GGU-psbD*) |
| 8 | 31257-31290 | 17 | 2.0 | 17 | 14 | 14 | 5 | 64 | IGS(*trnT-GGU-psbD*) |
| 9 | 30875-31554 | 340 | 2.0 | 340 | 32 | 14 | 16 | 36 | IGS(*trnT-GGU-psbD*) |
| 10 | 40779-40819 | 21 | 1.9 | 22 | 21 | 17 | 4 | 56 | IGS(*psaA-ycf3*) |
| 11 | 44625-44653 | 11 | 2.6 | 11 | 55 | 0 | 0 | 44 | IGS(*rps4-trnT-UGU*) |
| 12 | 54841-54928 | 5 | 18.2 | 5 | 64 | 0 | 3 | 31 | IGS(*rbcL-accD*) |
| 13 | 54841-54927 | 7 | 12.4 | 7 | 64 | 0 | 3 | 32 | IGS(*rbcL-accD*) |
| 14 | 54841-54915 | 34 | 2.2 | 35 | 64 | 0 | 4 | 32 | IGS(*rbcL-accD*) |
| 15 | 54848-54927 | 29 | 2.9 | 29 | 65 | 0 | 3 | 31 | IGS(*rbcL-accD*) |
| 16 | 56216-56256 | 15 | 2.7 | 15 | 31 | 0 | 46 | 21 | CDS(*accD*) |
| 17 | 56306-56498 | 15 | 11.7 | 15 | 22 | 8 | 34 | 33 | CDS(*accD*) |
| 18 | 56307-56453 | 51 | 2.9 | 51 | 22 | 8 | 34 | 34 | CDS(*accD*) |
| 19 | 56306-56498 | 66 | 2.9 | 64 | 22 | 8 | 34 | 33 | CDS(*accD*) |
| 20 | 57960-58156 | 98 | 2.0 | 99 | 33 | 14 | 23 | 28 | IGS-*ycf4* |
| 21 | 65517-65577 | 21 | 2.9 | 21 | 29 | 27 | 13 | 29 | IGS(*rpl33-rps18*) |
| 22 | 65753- 65947 | 95 | 2.0 | 96 | 38 | 17 | 18 | 24 | *rps18*-IGS |
| 23 | 72919-72959 | 21 | 1.9 | 22 | 48 | 4 | 12 | 34 | IGS(*psbH-petB*) |
| 24 | 88651-88712 | 18 | 3.4 | 18 | 25 | 9 | 35 | 29 | CDS(*ycf2*) |
| 25 | 92181-92241 | 31 | 2.0 | 31 | 29 | 9 | 21 | 39 | IGS(*5rrn-4.5rrn*) |
| 26 | 99412-99444 | 16 | 2.1 | 16 | 30 | 27 | 12 | 30 | IGS(*trnV-GAC-ycf15*) |
| 27 | 100033-100067 | 18 | 2.0 | 18 | 45 | 22 | 2 | 28 | IGS(*ycf15-rps12*) |
| 28 | 100037-100089 | 28 | 1.9 | 27 | 41 | 22 | 7 | 28 | IGS(*ycf15-rps12*) |
| 29 | 104950-105024 | 37 | 2.0 | 37 | 28 | 37 | 13 | 21 | IGS(*trnN-GUU-petB*) |
| 30 | 107410-107444 | 15 | 2.3 | 15 | 62 | 5 | 20 | 11 | IGS(*ycf1-rps15*) |
| 31 | 108784-109155 | 185 | 2.0 | 185 | 37 | 12 | 13 | 36 | IGS(*ycf1-rps15*) |
| 32 | 110228-110255 | 12 | 2.3 | 12 | 60 | 7 | 7 | 25 | IGS(*ycf1-rps15*) |
| 33 | 121447-121485 | 20 | 2.0 | 20 | 56 | 25 | 0 | 17 | CDS(*ndhF*) |
| 34 | 124674-124698 | 12 | 2.1 | 12 | 68 | 0 | 0 | 32 | CDS(*ccsA*) |
| 35 | 133947-133974 | 12 | 2.3 | 12 | 25 | 7 | 7 | 60 | IGS(*rps15-ycf1*) |
| 36 | 135047-135418 | 185 | 2.0 | 185 | 36 | 13 | 12 | 37 | IGS(*rps15-ycf1*) |
| 37 | 136758-136793 | 15 | 2.4 | 15 | 11 | 22 | 5 | 61 | IGS(*rps15-ycf1*) |
| 38 | 139178-139252 | 37 | 2.0 | 37 | 21 | 13 | 37 | 28 | IGS(*petB-trnN-GUU*) |
| 39 | 144113-144165 | 28 | 1.9 | 27 | 28 | 7 | 22 | 41 | IGS(*rps12-ycf15*) |
| 40 | 144135-144169 | 18 | 2.0 | 18 | 28 | 2 | 22 | 45 | IGS(*rps12-ycf15*) |
| 41 | 144758-144790 | 16 | 2.1 | 16 | 30 | 12 | 27 | 30 | IGS(*ycf15-trnV-GAC*) |
| 42 | 151961-152021 | 31 | 2.0 | 31 | 39 | 21 | 9 | 29 | IGS(*trnR-ACG-trnL-CAA*) |
| 43 | 155490-155551 | 18 | 3.4 | 18 | 29 | 35 | 9 | 25 | CDS(*ycf2*) |

**Table S7. Forward and palindromic repeats in *P. ovata* plastome.**

| **Repeat type** | **Repeat size** | **Repeat position 1** | **Repeat location 1** | **Repeat position 2** | **Repeat location 2** |
| --- | --- | --- | --- | --- | --- |
| F | 532 | 67470 | IGS(*rps12*-*clpP*) | 68037 | CDS*(clpP)* |
| F | 514 | 48762 | IGS(*ndhC-trnV-UAC*) | 49282 | *trnV-UAC*-IGS |
| F | 511 | 83780 | IGS(*rpl23-trnI-CAU*) | 84338 | IGS-*trnI-CAU* |
| F | 511 | 159352 | IGS-*TrnI-CAU* | 159910 | *trnI*-CAU- *rpl23* |
| F | 331 | 30883 | IGS(*trnT-psbD*) | 31223 | IGS(*trnT-psbD*) |
| F | 319 | 30895 | IGS(*trnT-psbD*) | 31235 | IGS(*trnT-psbD*) |
| F | 266 | 71571 | IGS(*psbB-trnL-CAA*) | 72073 | IGS(*psbT-psbN*) |
| F | 253 | 105828 | CDS(*ycf1*) | 119518 | IGS(*ndhD- ndhF*) |
| F | 200 | 30038 | IGS-*trnM-CAU*-IGS | 30311 | IGS-*trnM*-CAU-IGS |
| F | 187 | 108783 | IGS(*ycf1-rps15*) | 108968 | IGS(*ycf1-rps15*) |
| F | 187 | 135046 | IGS(rp*s15-ycf1*) | 135231 | IGS(*rps15-ycf1*) |
| F | 186 | 30052 | IGS-*trnM-CAU*-IGS | 30325 | IGS-*trnM-CAU*-IGS |
| F | 171 | 19792 | IGS(*rpoC2-rpoC1*) | 19999 | IGS(*rpoC2-rpoC1*) |
| F | 95 | 65757 | CDS(*rps18*) | 65852 | IGS-*rpl20* |
| F | 84 | 57959 | IGS(*psaI-ycf4*) | 58057 | IGS-*ycf4* |
| F | 73 | 23024 | CDS(*rpoB*) | 157716 | CDS(*ycf2*) |
| F | 73 | 65779 | CDS(*clpP*) | 65874 | CDS(*clpP*) |
| F | 72 | 74124 | CDS(*petB*) | 105319 | CDS(*petB*) |
| F | 71 | 71843 | IGS(*psbB-trnL-CAA*) | 90732 | *trnL-CAA* |
| F | 51 | 5815 | CDS(*rps16)* | 138958 | IGS(*petB-trnN*-GUU) |
| F | 44 | 56357 | CDS(*accD*) | 56423 | CDS(*accD*) |
| F | 47 | 56334 | CDS(*accD*) | 56451 | CDS(*accD*) |
| F | 42 | 56339 | CDS(*accD*) | 56456 | CDS(*accD*) |
| F | 41 | 106903 | IGS(*ycf1-rps15*) | 106995 | IGS(*ycf1-rps15*) |
| F | 41 | 137165 | IGS(*rps15-ycf1*) | 137257 | IGS(*rps15-ycf1*) |
| F | 43 | 100708 | IGS(*ycf15-rps12*) | 114600 | CDS(*ndhA*) |
| F | 43 | 129558 | CDS(*ndhA*) | 143450 | IGS(*rps12-ycf15*) |
| F | 38 | 71489 | IGS(*psbB-trnL-CAA*) | 71978 | IGS(*trnL-CA-psbT*) |
| F | 38 | 139177 | IGS(*petB-trnN-GUU*) | 139214 | IGS(*petB-trnN*-GUU) |
| F | 37 | 28432 | IGS(*petN-psbM*) | 28450 | IGS(*petN-psbM*) |
| F | 40 | 65516 | IGS(*rpl33-rsp18*) | 65537 | IGS(*rpl33-rsp18*) |
| F | 35 | 84249 | IGS(*trnI-CAU-trnI-CAU*) | 84924 | IGS(*trnI-CAU-trnI-CAU*) |
| p | 511 | 83780 | IGS(*rpl23 -trnI-CAU*) | 159352 | IGS-*trnI-CAU* |
| p | 511 | 84338 | IGS(*trnI-CAU-trnI-CAU*) | 159910 | IGS(*trnI-CAU-trnI-CAU*) |
| p | 253 | 119518 | IGS(*ndhD-ndhF*) | 138120 | CDS(*ycf1*) |
| p | 187 | 108783 | IGS(*rps15-ycf1*) | 135046 | IGS*(rps15-ycf1*) |
| p | 187 | 108968 | IGS(*rps15-ycf1*) | 135046 | IGS(*rps15-ycf1*) |
| p | 73 | 23024 | CDS(*rpoB*) | 86412 | CDS(*rpoB*) |
| p | 72 | 74124 | CDS(*petB*) | 138810 | CDS(*petB*) |
| p | 71 | 71843 | *trnL-CAA* | 153398 | *trnL-CAA* |
| p | 51 | 5815 | CDS(*rps16*) | 105192 | *trnN-GUU-* IGS |
| p | 41 | 106903 | IGS(*ycf1-rps15*) | 137165 | IGS(*rps15-ycf1*) |
| p | 41 | 106995 | IGS(*ycf1-rps15*) | 137257 | IGS(*rps15-ycf1*) |
| p | 43 | 100708 | IGS(*ycf15-rps12*) | 129558 | CDS(*ndhA*) |
| p | 43 | 114600 | CDS(*ndhA*) | 143450 | IGS(*rps12-ycf15*) |
| p | 38 | 104949 | IGS(*trnN-GUU-petB*) | 139177 | IGS(*petB-trnN-GUU*) |
| p | 38 | 104986 | IGS(*trnN-petB*) | 139214 | IGS(*petB- trnN-GUU*) |
| P | 35 | 84249 | IGS(*trnI-CAU- trnI-CAU*) | 159242 | IGS(*ycf2-trnI-CAU* |


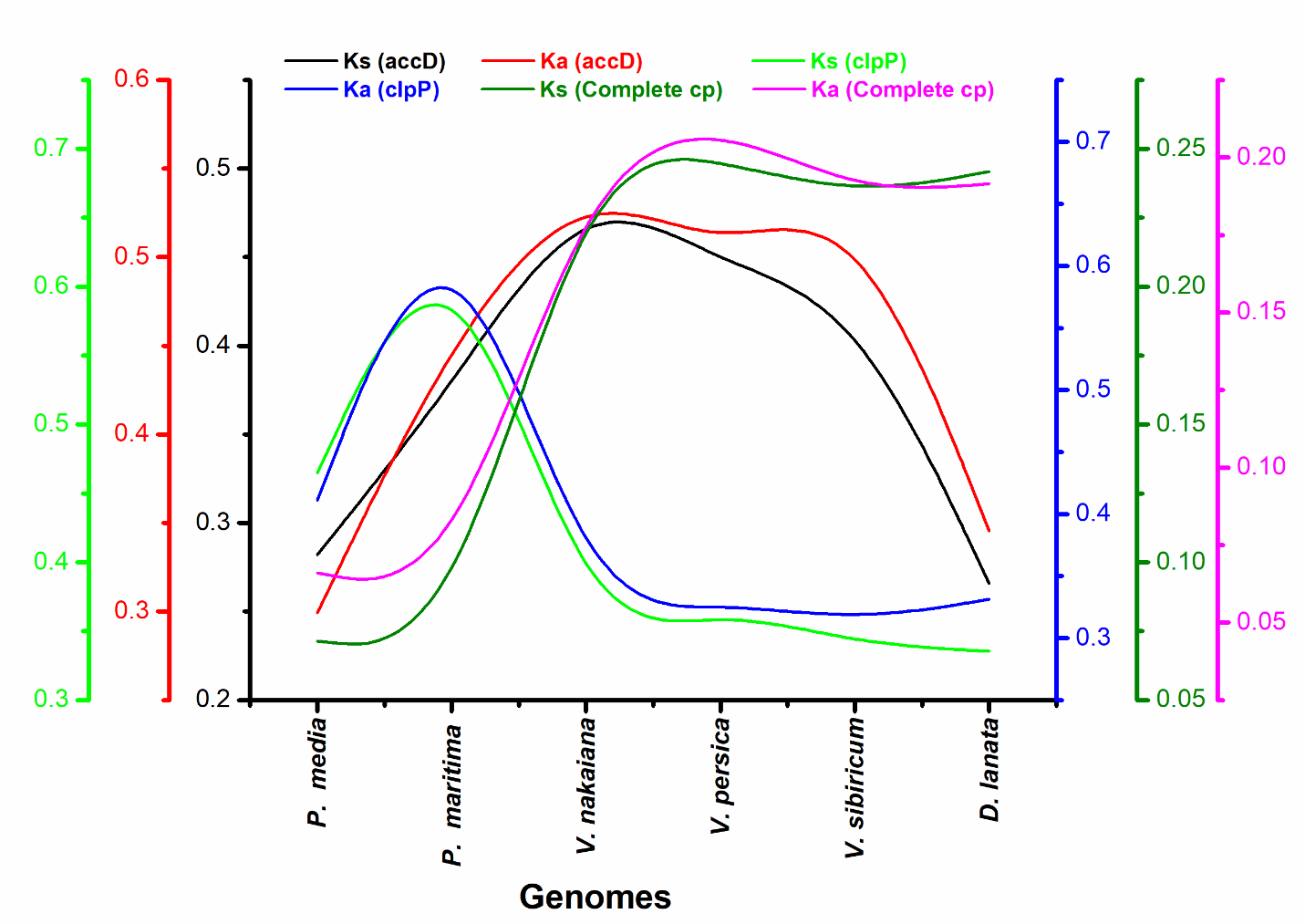


**Figure S1**. Ka, ks values of the *accD*, *clpP* and complete plastome of the chloroplast genomes of the P. ovata with related species.


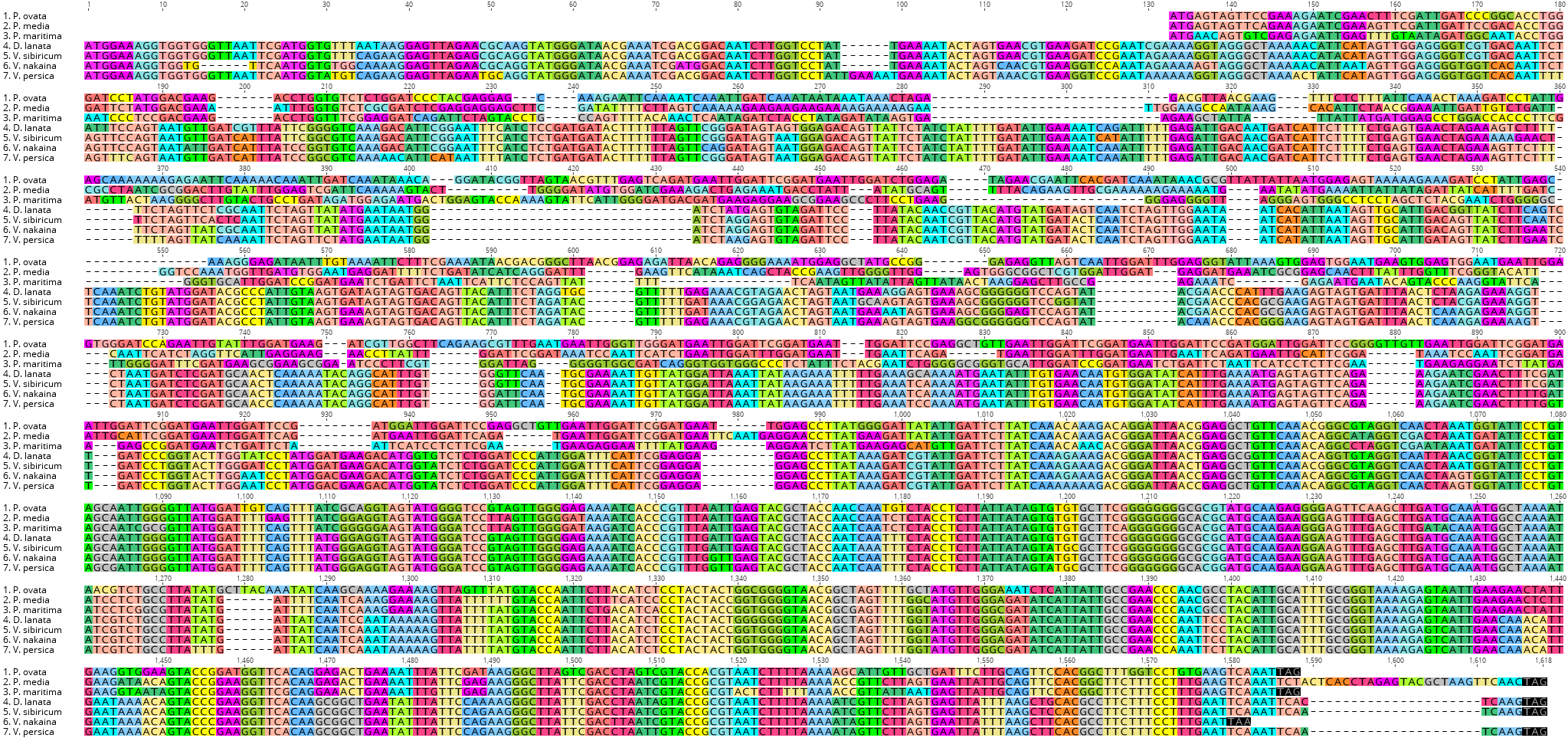


**Figure S2**. Alignment of *accD* gene nucleotide sequences among 7 *Plantaganaceae* species plastomes.


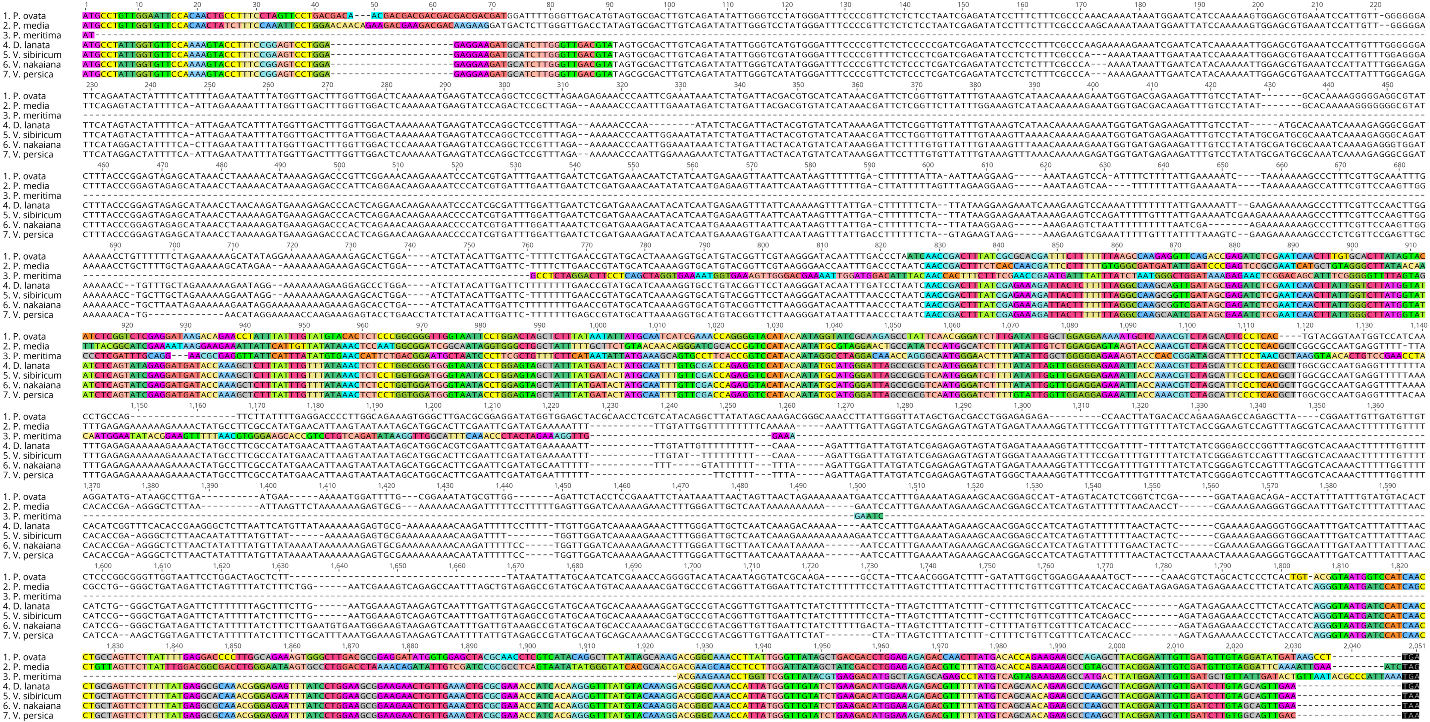


**Figure S3**. Alignment of *clpP* gene nucleotide sequences among 7 *Plantaganaceae* species plastomes.


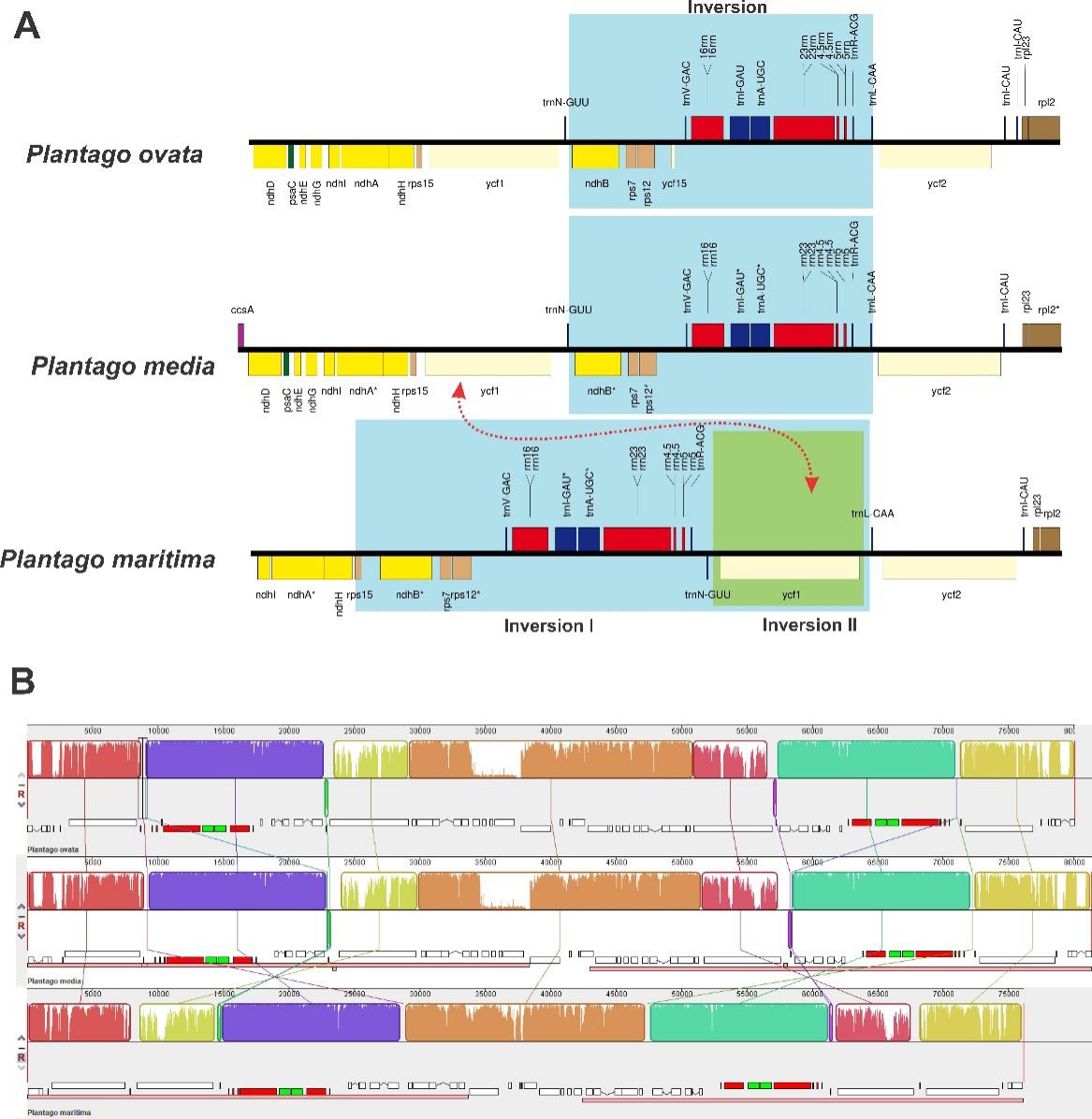


**Figure S4**. Alignment visualization of the IR regions among *P. ovata*, *P. media* and *P. maritima* plastome genomes. **(A)** IR regions are aligned and drawn with the OGDRAW online tool ((http://ogdraw.mpimp-golm.mpg.de) and large inversion regions are indicated in blue color. Green color shows additional inversion found only in *P. maritima* IR region. **(B)** MAUVE alignment of the IR regions of *Plantago* plastome sequences. *P. ovata* IR region was used as a reference.


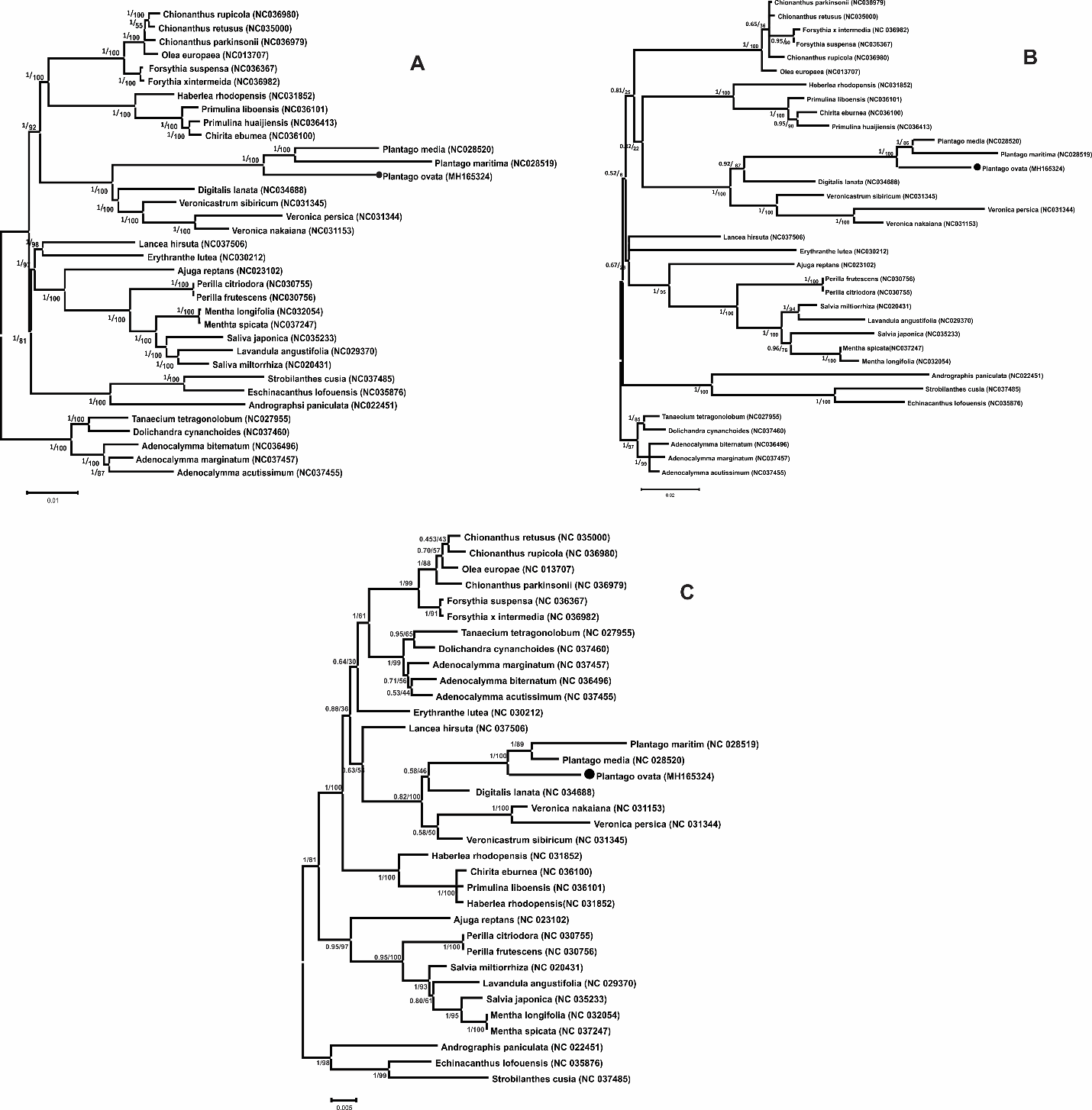


**Figure S5.** Phylogenetic trees were constructed for thirty-five species from eight families representing 22 genera using different methods, and tree is shown for the 72 protein coding genes **(A)**, *matK* **(B)** and *rbcL* **(C)** data sets. These sequences data sets were used with two different methods, Bayesian inference (BI) and maximum likelihood (ML). Numbers above the branches are the posterior probabilities of BI and bootstrap values of ML respectively. Black dots represent the position for *P. ovata.*

*
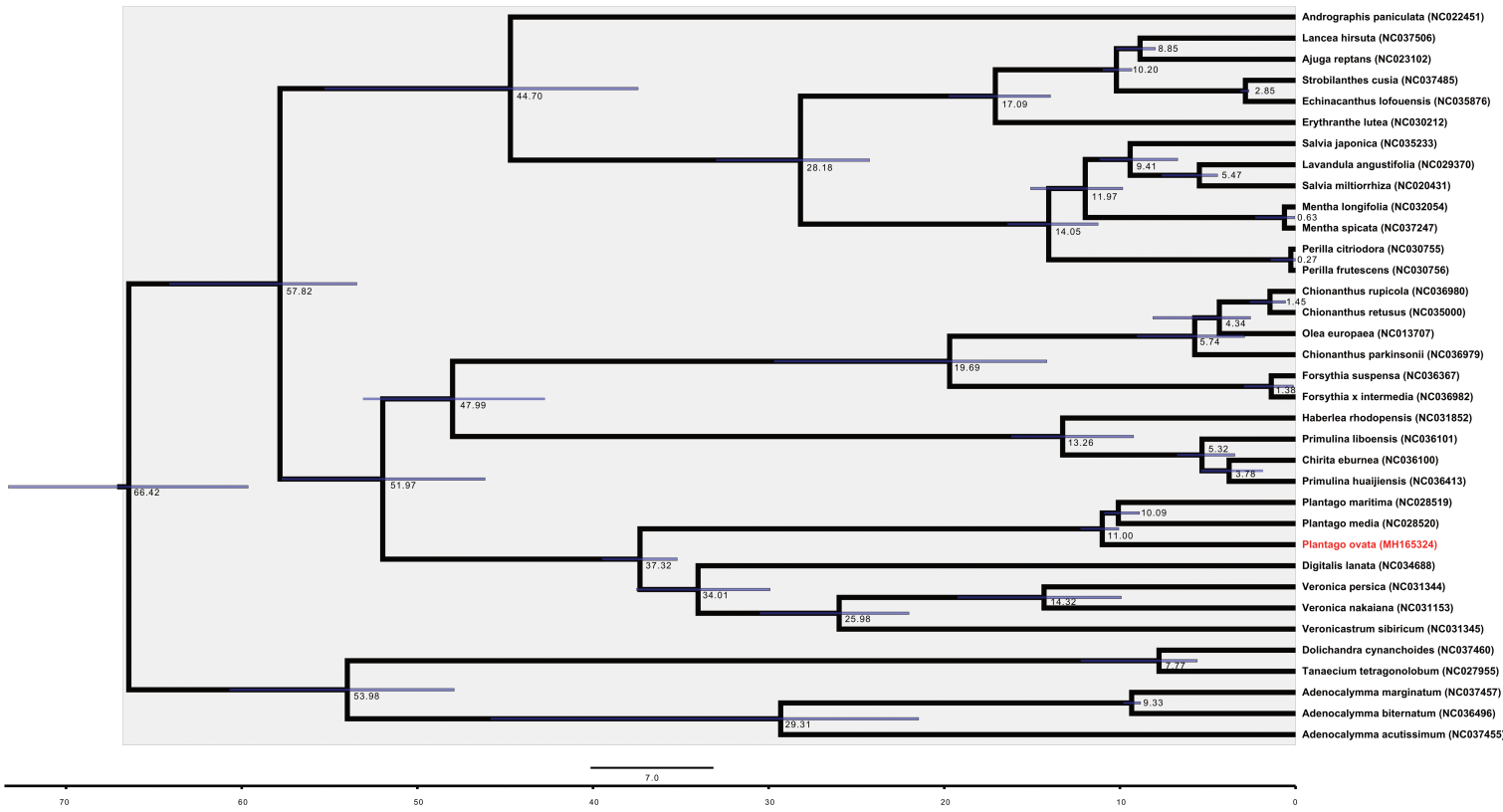
*

**Figure S6**. Bayesian chronogram of divergence time for *Plantago* species as employed in BEAST 1.8.5 [^106^](#_ENREF_109) rooted with family Bignoniaceae (*Dolichandra cyanachoides*, *Tanaecium tetragonolobum*, *Adenocalymma acutissimum*, *Adenocalymma biternatum* and *Adenocalymma marginatum*). General time reversible (GTR + G) substitution model was used with four rate categories, and a Yule tree speciation model with lognormal relaxed clock model in BEAST. The number near the nodes represent divergence time in million years, blue bar indicating 95% highest posterior densities.
