# Supplementary figures and images for: Expanded inverted repeat region with large scale inversion in the first complete plastid genome sequence of Plantago ovata
Source: Sci Rep. 2020 Mar 3;10:3881. doi: 10.1038/s41598-020-60803-y (PMC7054531; doi:10.1038/s41598-020-60803-y)

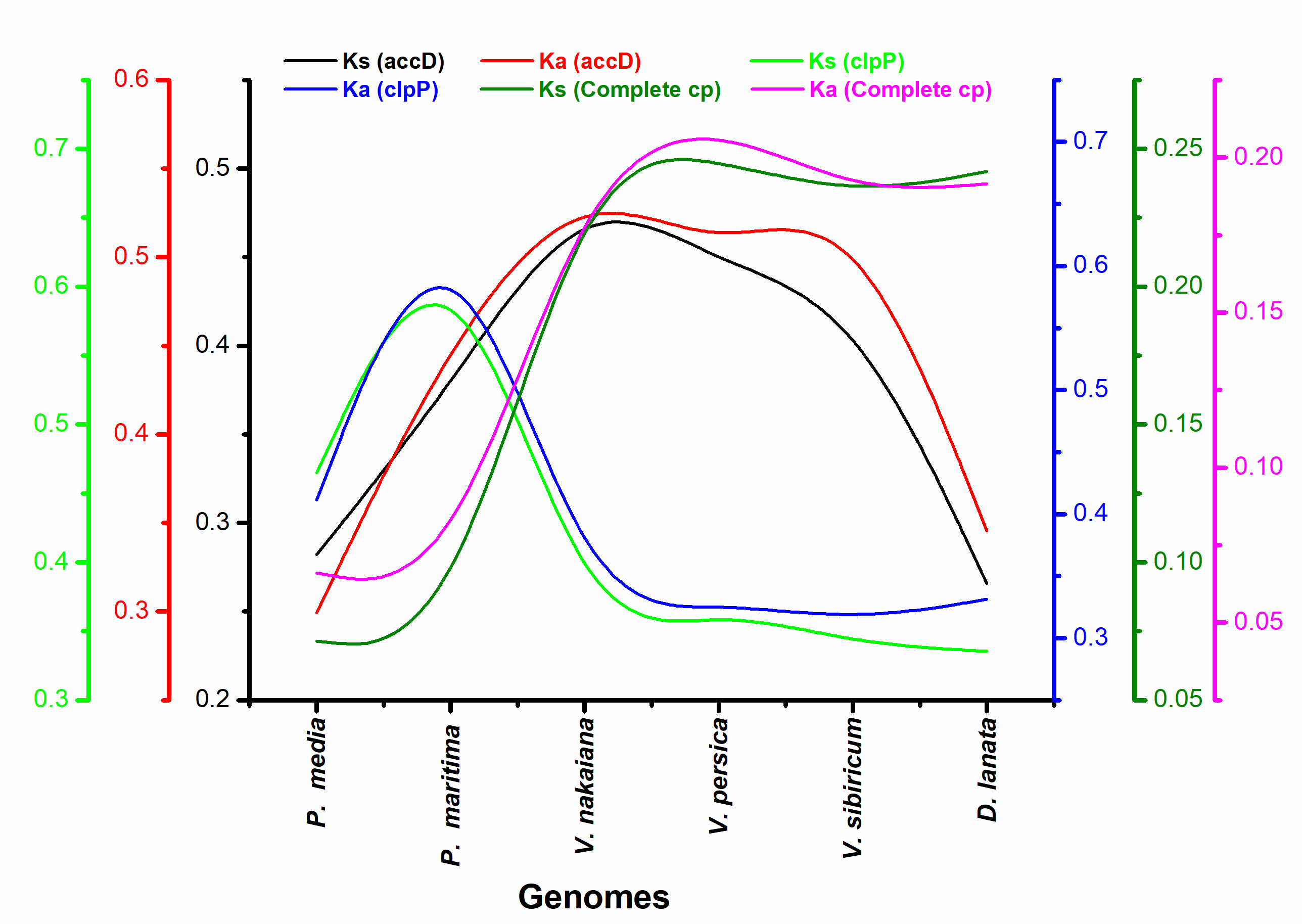

Supplement: Supplementary file 2 — Supplementary data set. [file 41598_2020_60803_MOESM2_ESM.zip › Supplementary data set/Figure S1.tif]

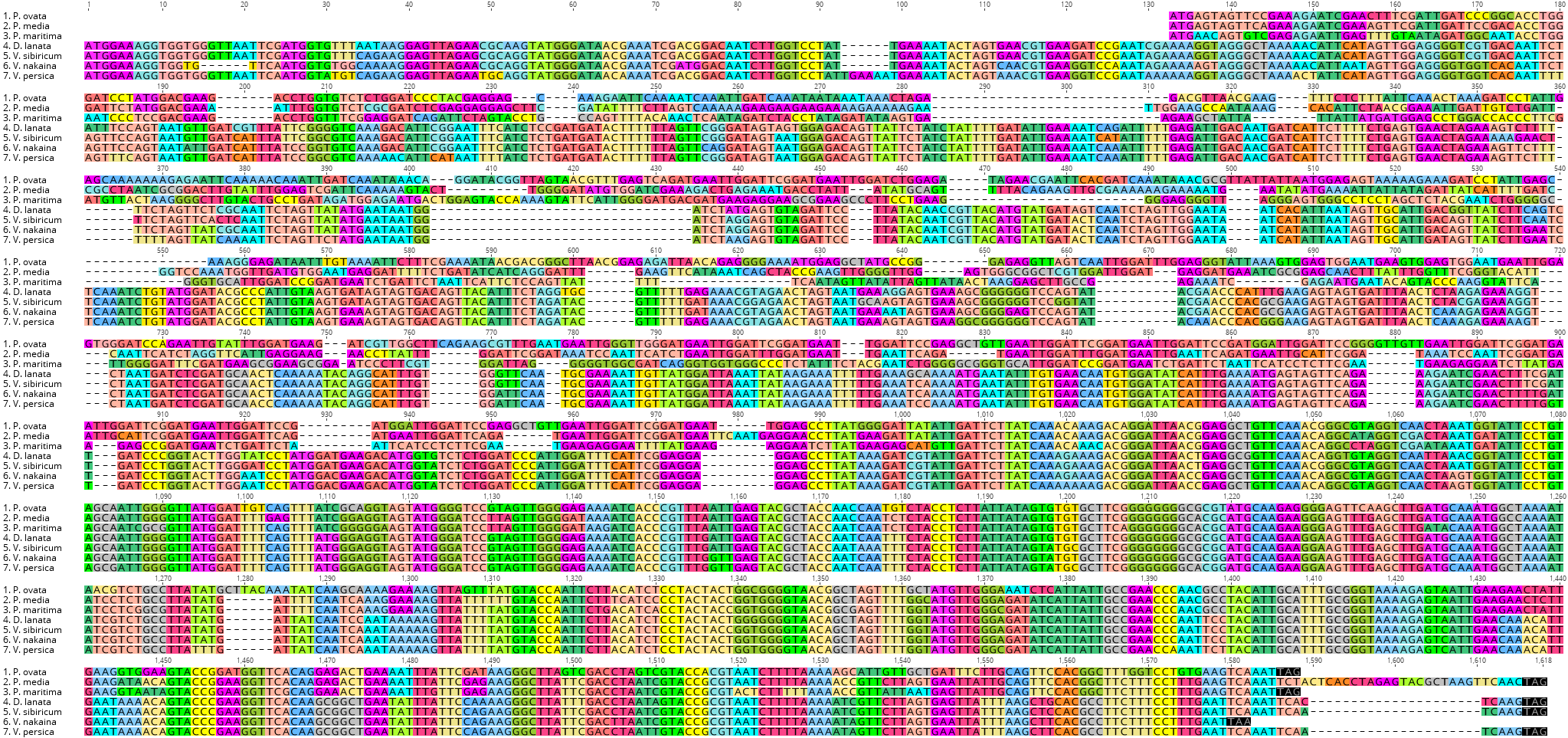

Supplement: Supplementary file 2 — Supplementary data set. [file 41598_2020_60803_MOESM2_ESM.zip › Supplementary data set/Figure S2.png]

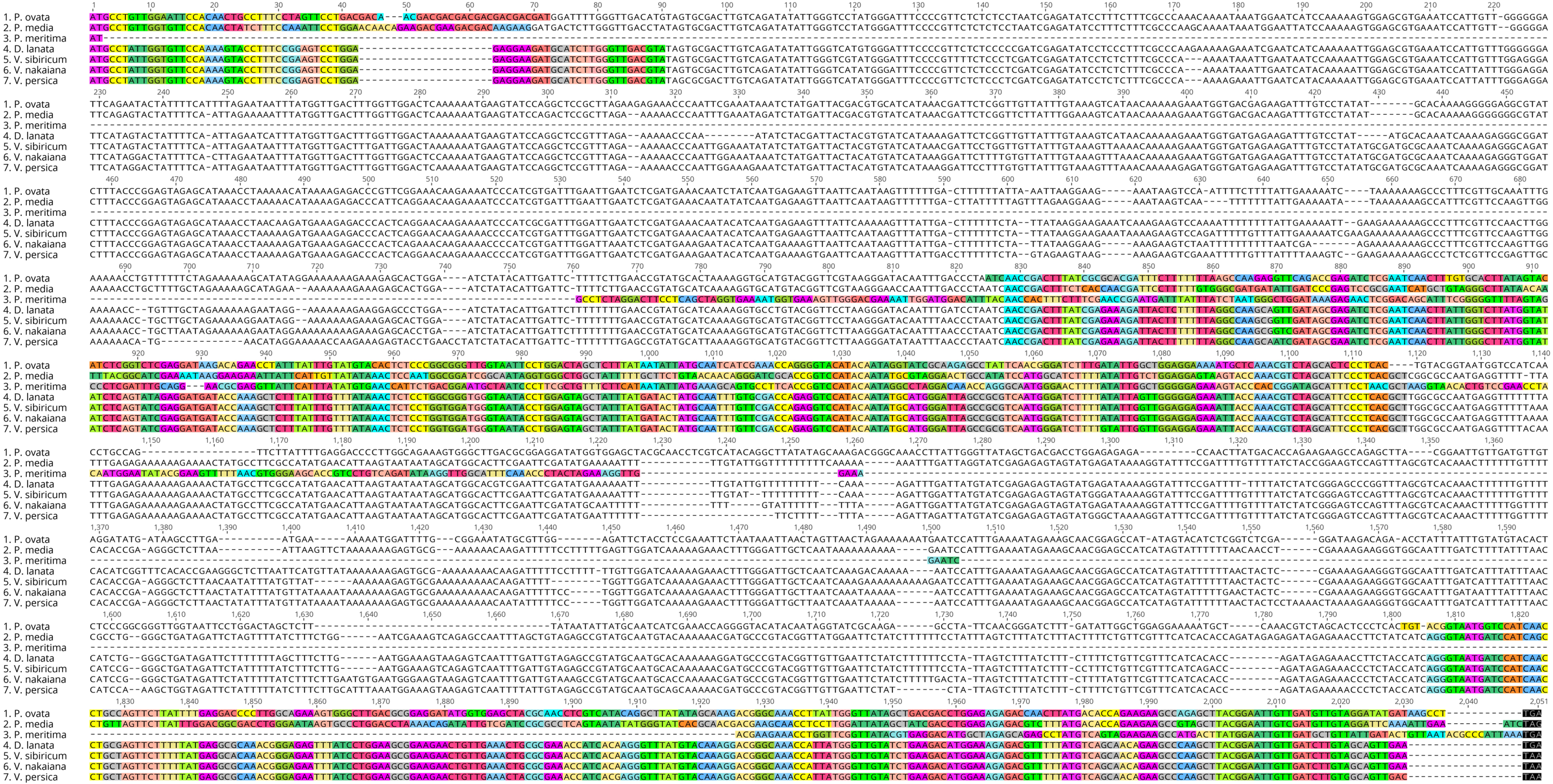

Supplement: Supplementary file 2 — Supplementary data set. [file 41598_2020_60803_MOESM2_ESM.zip › Supplementary data set/Figure S3.tif]

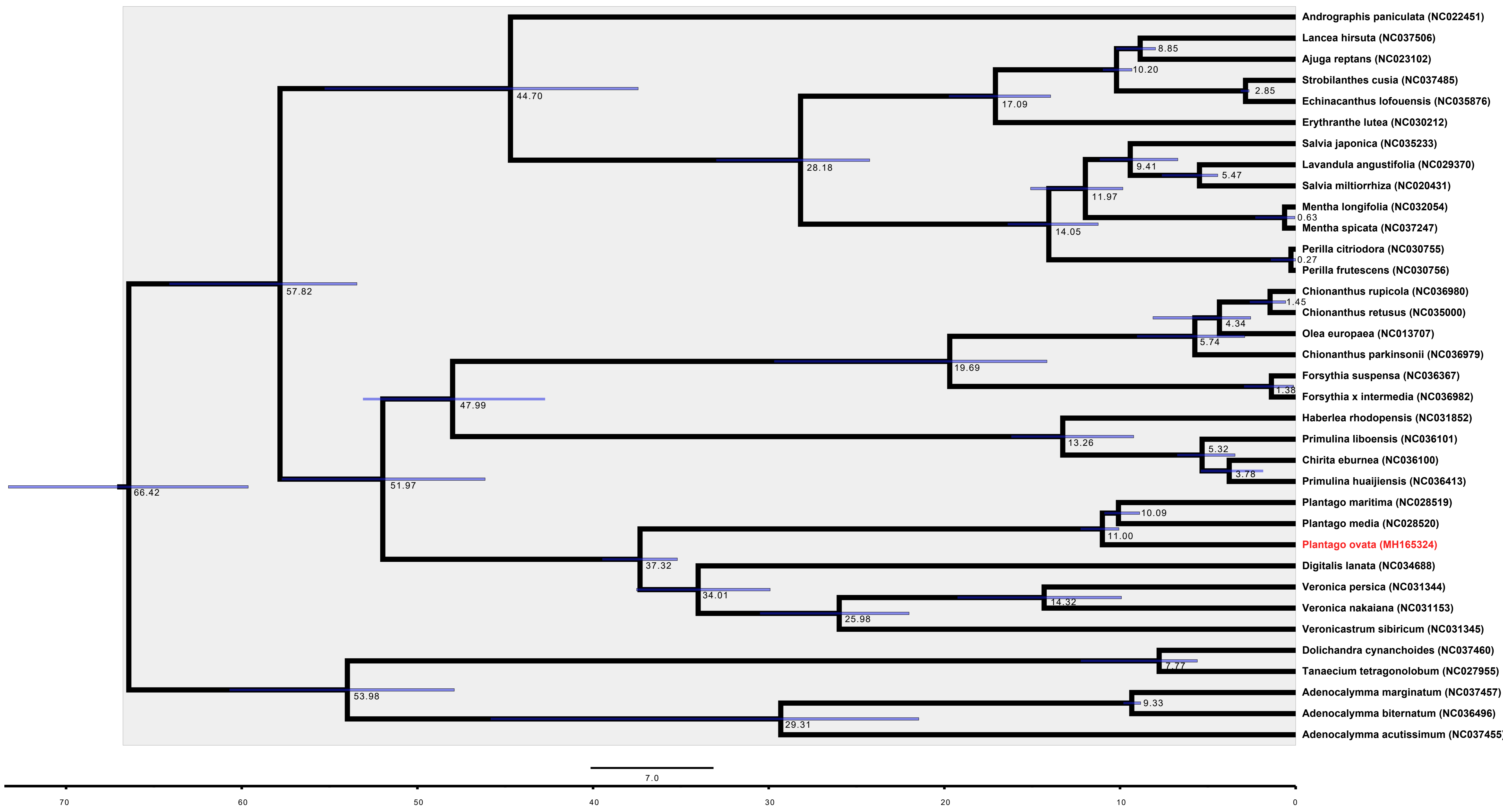

Supplement: Supplementary file 2 — Supplementary data set. [file 41598_2020_60803_MOESM2_ESM.zip › Supplementary data set/Figure S6.pdf]
